# MIBiG 4.0: advancing biosynthetic gene cluster curation through global collaboration

**DOI:** 10.1093/nar/gkae1115

**Published:** 2024-12-09

**Authors:** Mitja M Zdouc, Kai Blin, Nico L L Louwen, Jorge Navarro, Catarina Loureiro, Chantal D Bader, Constance B Bailey, Lena Barra, Thomas J Booth, Kenan A J Bozhüyük, José D D Cediel-Becerra, Zachary Charlop-Powers, Marc G Chevrette, Yit Heng Chooi, Paul M D’Agostino, Tristan de Rond, Elena Del Pup, Katherine R Duncan, Wenjia Gu, Novriyandi Hanif, Eric J N Helfrich, Matthew Jenner, Yohei Katsuyama, Aleksandra Korenskaia, Daniel Krug, Vincent Libis, George A Lund, Shrikant Mantri, Kalindi D Morgan, Charlotte Owen, Chin-Soon Phan, Benjamin Philmus, Zachary L Reitz, Serina L Robinson, Kumar Saurabh Singh, Robin Teufel, Yaojun Tong, Fidele Tugizimana, Dana Ulanova, Jaclyn M Winter, César Aguilar, Daniel Y Akiyama, Suhad A A Al-Salihi, Mohammad Alanjary, Fabrizio Alberti, Gajender Aleti, Shumukh A Alharthi, Mariela Y Arias Rojo, Amr A Arishi, Hannah E Augustijn, Nicole E Avalon, J Abraham Avelar-Rivas, Kyle K Axt, Hellen B Barbieri, Julio Cesar J Barbosa, Lucas Gabriel Barboza Segato, Susanna E Barrett, Martin Baunach, Christine Beemelmanns, Dardan Beqaj, Tim Berger, Jordan Bernaldo-Agüero, Sandra M Bettenbühl, Vincent A Bielinski, Friederike Biermann, Ricardo M Borges, Rainer Borriss, Milena Breitenbach, Kevin M Bretscher, Michael W Brigham, Larissa Buedenbender, Brodie W Bulcock, Carolina Cano-Prieto, João Capela, Victor J Carrion, Riley S Carter, Raquel Castelo-Branco, Gabriel Castro-Falcón, Fernanda O Chagas, Esteban Charria-Girón, Ayesha Ahmed Chaudhri, Vasvi Chaudhry, Hyukjae Choi, Yukyung Choi, Roya Choupannejad, Jakub Chromy, Melinda S Chue Donahey, Jérôme Collemare, Jack A Connolly, Kaitlin E Creamer, Max Crüsemann, Andres Arredondo Cruz, Andres Cumsille, Jean-Felix Dallery, Luis Caleb Damas-Ramos, Tito Damiani, Martinus de Kruijff, Belén Delgado Martín, Gerardo Della Sala, Jelle Dillen, Drew T Doering, Shravan R Dommaraju, Suhan Durusu, Susan Egbert, Mark Ellerhorst, Baptiste Faussurier, Artem Fetter, Marc Feuermann, David P Fewer, Jonathan Foldi, Andri Frediansyah, Erin A Garza, Athina Gavriilidou, Andrea Gentile, Jennifer Gerke, Hans Gerstmans, Juan Pablo Gomez-Escribano, Luz A González-Salazar, Natalie E Grayson, Claudio Greco, Juan E Gris Gomez, Sebastian Guerra, Shaday Guerrero Flores, Alexey Gurevich, Karina Gutiérrez-García, Lauren Hart, Kristina Haslinger, Beibei He, Teo Hebra, Jethro L Hemmann, Hindra Hindra, Lars Höing, Darren C Holland, Jonathan E Holme, Therese Horch, Pavlo Hrab, Jie Hu, Thanh-Hau Huynh, Ji-Yeon Hwang, Riccardo Iacovelli, Dumitrita Iftime, Marianna Iorio, Sidharth Jayachandran, Eunah Jeong, Jiayi Jing, Jung J Jung, Yuya Kakumu, Edward Kalkreuter, Kyo Bin Kang, Sangwook Kang, Wonyong Kim, Geum Jin Kim, Hyunwoo Kim, Hyun Uk Kim, Martin Klapper, Robert A Koetsier, Cassandra Kollten, Ákos T Kovács, Yelyzaveta Kriukova, Noel Kubach, Aditya M Kunjapur, Aleksandra K Kushnareva, Andreja Kust, Jessica Lamber, Martin Larralde, Niels J Larsen, Adrien P Launay, Ngoc-Thao-Hien Le, Sarah Lebeer, Byung Tae Lee, Kyungha Lee, Katherine L Lev, Shu-Ming Li, Yong-Xin Li, Cuauhtémoc Licona-Cassani, Annette Lien, Jing Liu, Julius Adam V Lopez, Nataliia V Machushynets, Marla I Macias, Taifo Mahmud, Matiss Maleckis, Añadir Maharai Martinez-Martinez, Yvonne Mast, Marina F Maximo, Christina M McBride, Rose M McLellan, Khyati Mehta Bhatt, Chrats Melkonian, Aske Merrild, Mikko Metsä-Ketelä, Douglas A Mitchell, Alison V Müller, Giang-Son Nguyen, Hera T Nguyen, Timo H J Niedermeyer, Julia H O’Hare, Adam Ossowicki, Bohdan O Ostash, Hiroshi Otani, Leo Padva, Sunaina Paliyal, Xinya Pan, Mohit Panghal, Dana S Parade, Jiyoon Park, Jonathan Parra, Marcos Pedraza Rubio, Huong T Pham, Sacha J Pidot, Jörn Piel, Bita Pourmohsenin, Malik Rakhmanov, Sangeetha Ramesh, Michelle H Rasmussen, Adriana Rego, Raphael Reher, Andrew J Rice, Augustin Rigolet, Adriana Romero-Otero, Luis Rodrigo Rosas-Becerra, Pablo Y Rosiles, Adriano Rutz, Byeol Ryu, Libby-Ann Sahadeo, Murrel Saldanha, Luca Salvi, Eduardo Sánchez-Carvajal, Christian Santos-Medellin, Nicolau Sbaraini, Sydney M Schoellhorn, Clemens Schumm, Ludek Sehnal, Nelly Selem, Anjali D Shah, Tania K Shishido, Simon Sieber, Velina Silviani, Garima Singh, Hemant Singh, Nika Sokolova, Eva C Sonnenschein, Margherita Sosio, Sven T Sowa, Karin Steffen, Evi Stegmann, Alena B Streiff, Alena Strüder, Frank Surup, Tiziana Svenningsen, Douglas Sweeney, Judit Szenei, Azat Tagirdzhanov, Bin Tan, Matthew J Tarnowski, Barbara R Terlouw, Thomas Rey, Nicola U Thome, Laura Rosina Torres Ortega, Thomas Tørring, Marla Trindade, Andrew W Truman, Marie Tvilum, Daniel W Udwary, Christoph Ulbricht, Lisa Vader, Gilles P van Wezel, Max Walmsley, Randika Warnasinghe, Heiner G Weddeling, Angus N M Weir, Katherine Williams, Sam E Williams, Thomas E Witte, Steffaney M Wood Rocca, Keith Yamada, Dong Yang, Dongsoo Yang, Jingwei Yu, Zhenyi Zhou, Nadine Ziemert, Lukas Zimmer, Alina Zimmermann, Christian Zimmermann, Justin J J van der Hooft, Roger G Linington, Tilmann Weber, Marnix H Medema

**Affiliations:** Bioinformatics Group, Wageningen University & Research, Droevendaalsesteeg 1, 6708 PB Wageningen, The Netherlands; The Novo Nordisk Foundation Center for Biosustainability, Technical University of Denmark, Building 220, Søltofts Plads, 2800 Kongens Lyngby, Denmark; Bioinformatics Group, Wageningen University & Research, Droevendaalsesteeg 1, 6708 PB Wageningen, The Netherlands; Bioinformatics Group, Wageningen University & Research, Droevendaalsesteeg 1, 6708 PB Wageningen, The Netherlands; Bioinformatics Group, Wageningen University & Research, Droevendaalsesteeg 1, 6708 PB Wageningen, The Netherlands; Helmholtz Institute for Pharmaceutical Research Saarland (HIPS), Helmholtz Centre for Infection Research (HZI), Campus E8.1, 66123 Saarbrücken, Germany; School of Chemistry, Chemistry Building, University of Sydney, Eastern Ave, Camperdown NSW 2050, Sydney, New South Wales, Australia; Department of Chemistry, University of Konstanz, Universitätsstraße 10, 78464 Konstanz, Germany; The Novo Nordisk Foundation Center for Biosustainability, Technical University of Denmark, Building 220, Søltofts Plads, 2800 Kongens Lyngby, Denmark; Helmholtz Institute for Pharmaceutical Research Saarland (HIPS), Helmholtz Centre for Infection Research (HZI), Campus E8.1, 66123 Saarbrücken, Germany; Myria Biosciences AG, Tech Park Basel, Hochbergstrasse 60C, 4057 Basel, Switzerland; Department of Microbiology and Cell Science, University of Florida, 1355 Museum Drive, Gainesville, Florida, 32611, USA; Ginkgo Bioworks, 27 Drydock Avenue, 8th Floor, Boston, MA 02210, USA; Department of Microbiology and Cell Science, University of Florida, 1355 Museum Drive, Gainesville, Florida, 32611, USA; University of Florida Genetics Institute, University of Florida, 2033 Mowry Rd, Gainesville, FL 32611, USA; School of Molecular Sciences, University of Western Australia, 35 Stirling Highway, Perth 6009, Australia; Helmholtz Institute for Pharmaceutical Research Saarland (HIPS), Helmholtz Centre for Infection Research (HZI), Campus E8.1, 66123 Saarbrücken, Germany; Chair of Technical Biochemistry, Technical University of Dresden, Bergstraße 66, 01069 Dresden, Germany; School of Chemical Sciences, University of Auckland, 23 Symonds St, Auckland 1010, New Zealand; Bioinformatics Group, Wageningen University & Research, Droevendaalsesteeg 1, 6708 PB Wageningen, The Netherlands; Newcastle University, Biosciences Institute, Catherine Cookson Building, Newcastle upon Tyne, NE2 4HH, UK; Sutro Biopharma, 111 Oyster Point Blvd, South San Francisco, CA, 94080, USA; Department of Chemistry, Faculty of Mathematics and Natural Sciences, IPB University, Gedung Kimia Wing 1 Lantai 3, Jalan Tanjung Kampus IPB Dramaga, Bogor, Jawa Barat 16680, Indonesia; Institute for Molecular Bio Science, Goethe University Frankfurt, Max-von-Laue Strasse 9, 60438 Frankfurt am Main, Germany; LOEWE Center for Translational Biodiversity Genomics (TBG), Senckenberganlage 25, 60325 Frankfurt am Main, Germany; Senckenberg Society for Nature Research, Senckenberganlage 25, 60325 Frankfurt am Main, Germany; Department of Chemistry, University of Warwick, Gibbet Hill Rd, Coventry, CV4 7AL, UK; Warwick Integrative Synthetic Biology Centre (WISB), University of Warwick, Gibbet Hill Rd, Coventry, CV4 7AL, UK; Department of Biotechnology, Graduate School of Agricultural and Life Sciences, The University of Tokyo, 1-1-1, Yayoi, Bunkyo-ku, Tokyo, 113-8657, Japan; Collaborative Research Institute for Innovative Microbiology, The University of Tokyo, 1-1-1, Yayoi, Bunkyo-ku, Tokyo, 113-8657, Japan; Translational Genome Mining for Natural Products, Interfaculty Institute of Microbiology and Infection Medicine Tübingen (IMIT), Interfaculty Institute for Biomedical Informatics (IBMI), University of Tübingen, Sand 14, 72076 Tübingen, Germany; Helmholtz Institute for Pharmaceutical Research Saarland (HIPS), Helmholtz Centre for Infection Research (HZI), Campus E8.1, 66123 Saarbrücken, Germany; Saarland University, Campus E8.1, 66123 Saarbrücken, Germany; Department of Microbial Drugs, Helmholtz Centre for Infection Research (HZI), Inhoffenstr. 7, 38124 Braunschweig, Germany; Université Paris Cité - Inserm Unit 1284, 75015 Paris, France; Translational Genome Mining for Natural Products, Generare Bioscience, 75011 Paris, Île-de-France, France; Sustainable Soils and Crops, Rothamsted Research, West Common, Harpenden, Hertfordshire, AL5 2JQ, UK; Computational Biology Lab, National Agri-Food and Biomanufacturing Institute (NABI), Sector 81, S.A.S. Nagar, Mohali, Punjab 140306, India; Regional Centre for Biotechnology, NCR Biotech Science Cluster, 3rd Milestone, Faridabad-Gurugram Expressway, Faridabad - 121001 Haryana (NCR Delhi), India; Department of Chemistry and Biochemistry, University of Northern British Columbia, 3333 University Way, Prince George, BC, V2N 4Z9, Canada; Biochemistry and Metabolism, John Innes Centre, Norwich Research Park, Colney Lane, Norwich, NR4 7UH, UK; Latvian Institute of Organic Synthesis, Aizkraukles street 21, LV-1006 Riga, Latvia; Department of Pharmaceutical Sciences, Oregon State University, 1601 SW Jefferson Way, Corvallis, OR 97331-3507, USA; Department of Ecology, Evolution and Marine Biology, University of California, 1169 Biological Sciences II, Santa Barbara, CA 93106, USA; Department of Environmental Microbiology, Swiss Federal Institute of Aquatic Science and Technology, Ueberlandstrasse 133, 8600 Duebendorf, Switzerland; Bioinformatics Group, Wageningen University & Research, Droevendaalsesteeg 1, 6708 PB Wageningen, The Netherlands; Plant-Microbe Interactions, Institute of Environmental Biology, Utrecht University, 3584 CH Utrecht, The Netherlands; Faculty of Environment, Science and Economy, University of Exeter, TR10 9FE, Penryn, Cornwall, UK; Department of Pharmaceutical Sciences, University of Basel, Klingelbergstrasse 50, 4056 Basel, Switzerland; State Key Laboratory of Microbial Metabolism, School of Life Sciences and Biotechnology, Shanghai Jiao Tong University, Minhang District, Shanghai 200240, China; Department of Biochemistry, University of Johannesburg, C2 Lab Building 224, Kingsway Campus, Cnr University & Kingsway Road, Auckland Park, Johannesburg 2006, South Africa; International Research and Development (R&D) Division, Omnia Nutriology, Omnia Holdings Ltd, Building H, Monte Circle, 178 Montecasino Blvd, Fourways, Sandton, 2055, South Africa; Department of Marine Resource Science, Faculty of Agriculture and Marine Science, Kochi University, 200 Otsu, Monobe, Nankoku-shi, Kochi, 783-8502, Japan; Marine Core Research Institute, Kochi University, 200 Otsu, Monobe, Nankoku-shi, Kochi, 783-8502, Japan; Department of Pharmacology and Toxicology, University of Utah, 30 S 2000 E, Salt Lake City, Utah, 84112, USA; Industrial Genomics Laboratory, Centro de Biotecnología FEMSA, Escuela de Ingeniería y Ciencias, Tecnológico de Monterrey, Av. Eugenio Garza Sada 2501sur, Nuevo Leon, 64700, México; Department of Chemistry, Purdue University, 610 Purdue Mall, West Lafayette, IN, 47907, USA; Department of Organic Chemistry, Institute of Chemistry, University of Campinas (UNICAMP), Rua Monteiro Lobato 270, Campinas, São Paulo, 13.083-862, Brazil; Department of Applied Sciences, University of Technology, Al-Sina’a St., 10066, Baghdad, Iraq; Bioinformatics Group, Wageningen University & Research, Droevendaalsesteeg 1, 6708 PB Wageningen, The Netherlands; School of Life Sciences, University of Warwick, Gibbet Hill Road, Coventry, CV4 7AL, UK; Department of Food and Animal Sciences, College of Agriculture, Tennessee State University, Nashville, TN 37209, USA; Department of Biosciences, Swansea University, Singleton Park, Swansea, SA2 8PP, UK; Escuela Nacional de Estudios Superiores Unidad Leon, Universidad Nacional Autonoma de Mexico, Blv. UNAM #2011, Predio El Saucillo y, Comunidad de los Tepetates, El Potrero, 37684 León de los Aldama, Gto., Mexico; School of Molecular Sciences, University of Western Australia, 35 Stirling Highway, Perth 6009, Australia; Department of Botany and Microbiology, College of Science, King Saud University, P.O. Box 2455, Riyadh, 11451, Saudi Arabia; Bioinformatics Group, Wageningen University & Research, Droevendaalsesteeg 1, 6708 PB Wageningen, The Netherlands; Institute of Biology, Leiden University, Sylviusweg 72, 2333BE Leiden, The Netherlands; Center for Marine Biotechnology and Biomedicine, Scripps Institution of Oceanography, University of California San Diego, 9500 Gilman Drive, La Jolla, CA 92093-0212, USA; Unidad de Genómica Avanzada, Centro de Investigación y de Estudios Avanzados del Instituto Politécnico Nacional, Km 9.6 Libramiento Norte Carretera Irapuato-León, 36824, Irapuato, Mexico; Department of Pharmaceutical Sciences, Oregon State University, 1601 SW Jefferson Way, Corvallis, OR 97331-3507, USA; Department of Organic Chemistry, Institute of Chemistry, University of Campinas (UNICAMP), Rua Monteiro Lobato 270, Campinas, São Paulo, 13.083-862, Brazil; Department of Organic Chemistry, Institute of Chemistry, University of Campinas (UNICAMP), Rua Monteiro Lobato 270, Campinas, São Paulo, 13.083-862, Brazil; Department of Chemistry, University of Manitoba, 66 Chancellors Circle, Winnipeg, MB R3T 2N2, Canada; Department of Chemistry, University of Illinois Urbana-Champaign, 600 S. Matthews Ave, Urbana, IL 61801, USA; Carl R. Woese Institute for Genomic Biology, University of Illinois Urbana-Champaign, 1206 W. Gregory Drive, Urbana, IL 61801, USA; Institute of Pharmaceutical Biology, University of Bonn, Nussallee 6, 53115 Bonn, Germany; Helmholtz Institute for Pharmaceutical Research Saarland (HIPS), Helmholtz Centre for Infection Research (HZI), Campus E8.1, 66123 Saarbrücken, Germany; Saarland University, Campus E8.1, 66123 Saarbrücken, Germany; Interfaculty Institute of Microbiology and Infection Medicine Tübingen, Microbial Bioactive Compounds, University of Tübingen, Auf der Morgenstelle 28, 72076, Tübingen, Germany; Institute of Pharmaceutical Biology and Biotechnology, University of Marburg, Robert-Koch-Str. 4, 35037 Marburg, Germany; Departamento de Microbiología Molecular, Instituto de Biotecnología, Universidad Nacional Autónoma de México, Av. Universidad 2001, Chamilpa, 62210 Cuernavaca, Morelos, México; Facultad de Ciencias Biológicas, Universidad Nacional Mayor de San Marcos, Av. Germán Amézaga S/N, Ciudad Universitaria, UNMSM, Lima 15081, Perú; Institute for Molecular Bio Science, Goethe University Frankfurt, Max-von-Laue Strasse 9, 60438 Frankfurt am Main, Germany; LOEWE Center for Translational Biodiversity Genomics (TBG), Senckenberganlage 25, 60325 Frankfurt am Main, Germany; Departamento de Bioquímica, Instituto de Química, Universidade Federal do Rio de Janeiro, Avenida Athos da Silveira Ramos, no 149, Rio de Janeiro, 21941-909, Brazil; Bioinformatics Group, Wageningen University & Research, Droevendaalsesteeg 1, 6708 PB Wageningen, The Netherlands; Institute for Molecular Bio Science, Goethe University Frankfurt, Max-von-Laue Strasse 9, 60438 Frankfurt am Main, Germany; LOEWE Center for Translational Biodiversity Genomics (TBG), Senckenberganlage 25, 60325 Frankfurt am Main, Germany; Instituto de Pesquisas de Produtos Naturais Walter Mors, Universidade Federal do Rio de Janeiro, CCS - Av. Carlos Chagas Filho, 373 - Bloco H - Cidade Universitária, Rio de Janeiro - RJ, 21941-599, Brazil; Department of Biology, Humboldt University Berlin, Invaliden-Str. 42, 10115 Berlin, Germany; Institute of Marine Biotechnology (IMaB), University of Greifswald,Walter-Rathenau-Str. 49A, 17489 Greifswald, Germany; Institute for Molecular Bio Science, Goethe University Frankfurt, Max-von-Laue Strasse 9, 60438 Frankfurt am Main, Germany; LOEWE Center for Translational Biodiversity Genomics (TBG), Senckenberganlage 25, 60325 Frankfurt am Main, Germany; Institute of Biology, Leiden University, Sylviusweg 72, 2333BE Leiden, The Netherlands; Department of Crop Protection, Instituto de Hortofruticultura Subtropical y Mediterránea “La Mayora” (IHSM-UMA-CSIC), Campus Universitario de Teatinos, 29010, Málaga, Spain; Department of Microbial Ecology, Netherlands Institute of Ecology (NIOO-KNAW), Droevendaalsesteeg 10 6708 PB Wageningen, The Netherlands; School of Cellular and Molecular Biology, Faculty of Biological Sciences, University of Leeds, 6 Clarendon Way, Woodhouse, Leeds, LS2 3AA, UK; CICA – Centro Interdisciplinar de Química e Bioloxía, Universidade da Coruña, As Carballeiras, s/n, Campus de Elviña, 15071 A Coruña, Spain; School of Molecular Sciences, University of Western Australia, 35 Stirling Highway, Perth 6009, Australia; The Novo Nordisk Foundation Center for Biosustainability, Technical University of Denmark, Building 220, Søltofts Plads, 2800 Kongens Lyngby, Denmark; Centre of Biological Engineering, University of Minho, 4710-057, Braga, Portugal; Institute of Biology, Leiden University, Sylviusweg 72, 2333BE Leiden, The Netherlands; Department of Crop Protection, Instituto de Hortofruticultura Subtropical y Mediterránea “La Mayora” (IHSM-UMA-CSIC), Campus Universitario de Teatinos, 29010, Málaga, Spain; Department of Microbiology, Faculty of Science, Campus Universitario de Teatinos s/n, University of Málaga, 29010 Málaga, Spain; Department of Chemistry, University of Illinois Urbana-Champaign, 600 S. Matthews Ave, Urbana, IL 61801, USA; Carl R. Woese Institute for Genomic Biology, University of Illinois Urbana-Champaign, 1206 W. Gregory Drive, Urbana, IL 61801, USA; Interdisciplinary Centre of Marine and Environmental Research (CIIMAR/CIMAR), University of Porto, 4450-208 Matosinhos, Portugal; Center for Marine Biotechnology and Biomedicine, Scripps Institution of Oceanography, University of California San Diego, 9500 Gilman Drive, La Jolla, CA 92093-0212, USA; Instituto de Pesquisas de Produtos Naturais Walter Mors, Universidade Federal do Rio de Janeiro, CCS - Av. Carlos Chagas Filho, 373 - Bloco H - Cidade Universitária, Rio de Janeiro - RJ, 21941-599, Brazil; Department of Microbial Drugs, Helmholtz Centre for Infection Research (HZI), Inhoffenstr. 7, 38124 Braunschweig, Germany; Institute of Microbiology, Technische Universität Braunschweig, Spielmannstraße 7, 38106 Braunschweig, Germany; Institute for Molecular Bio Science, Goethe University Frankfurt, Max-von-Laue Strasse 9, 60438 Frankfurt am Main, Germany; LOEWE Center for Translational Biodiversity Genomics (TBG), Senckenberganlage 25, 60325 Frankfurt am Main, Germany; Microbial Interactions in Plant Ecosystems, IMIT/ZMBP, Eberhard Karls University of Tübingen, 72076 Tübingen, Germany; College of Pharmacy, Yeungnam University, 280 Daehak-ro Gyeongsan-Si, Gyeongsangbuk-do 38541, Republic of Korea; Research Institute of Cell Culture, Yeungnam University, 280 Daehak-ro Gyeongsan-Si, Gyeongsangbuk-do 38541, Republic of Korea; College of Pharmacy and Drug Information Research Institute, Sookmyung Women's University, 100 Cheongpa-ro 47 gil, Seoul 04310, Republic of Korea; Fungal Natural Products, Westerdijk Fungal Biodiversity Institute, Uppsalalaan 8, 3584CT Utrecht, The Netherlands; Manchester Institute of Biotechnology, Department of Chemistry, School of Natural Sciences, Faculty of Science and Engineering, University of Manchester, 131 Princess Street, Manchester M1 7DN, UK; Department of Microbiology and Cell Science, University of Florida, 1355 Museum Drive, Gainesville, Florida, 32611, USA; Fungal Natural Products, Westerdijk Fungal Biodiversity Institute, Uppsalalaan 8, 3584CT Utrecht, The Netherlands; Manchester Institute of Biotechnology, Department of Chemistry, School of Natural Sciences, Faculty of Science and Engineering, University of Manchester, 131 Princess Street, Manchester M1 7DN, UK; Innovative Genomics Institute, University of California Berkeley, 2151Berkeley Way, Berkeley, CA 94720, USA; Department of Earth and Planetary Science, University of California, 307 McCone Hall Berkeley, CA 94720-4767, USA; Institute of Pharmaceutical Biology, University of Bonn, Nussallee 6, 53115 Bonn, Germany; Escuela Nacional de Estudios Superiores Unidad Leon, Universidad Nacional Autonoma de Mexico, Blv. UNAM #2011, Predio El Saucillo y, Comunidad de los Tepetates, El Potrero, 37684 León de los Aldama, Gto., Mexico; Department of Microbiology and Cell Science, University of Florida, 1355 Museum Drive, Gainesville, Florida, 32611, USA; Université Paris-Saclay, INRAE, UR BIOGER, 22 place de l’Agronomie, 91120 Palaiseau, France; The Novo Nordisk Foundation Center for Biosustainability, Technical University of Denmark, Building 220, Søltofts Plads, 2800 Kongens Lyngby, Denmark; IOCB Prague, Czech Academy of Science, Flemingovo náměstí 542/2, 160 00 Praha 6, Czech Republic; Helmholtz Institute for Pharmaceutical Research Saarland (HIPS), Helmholtz Centre for Infection Research (HZI), Campus E8.1, 66123 Saarbrücken, Germany; Department of Microbiology, Faculty of Science, Campus Universitario de Teatinos s/n, University of Málaga, 29010 Málaga, Spain; Department of Ecosustainable Marine Biotechnology, Stazione Zoologica Anton Dohrn, Giardini del Molosiglio, Via A.F. Acton 55, 80133 Naples, Italy; Department of Bioscience Engineering, Research Group Laboratory of Applied Microbiology and Biotechnology, University of Antwerp, Groenenborgerlaan 171, 2020 Antwerp, Belgium; US Department of Energy Joint Genome Institute, Lawrence Berkeley National Laboratory, 1 Cyclotron Road, Berkeley, CA 94720, USA; Department of Chemistry, University of Illinois Urbana-Champaign, 600 S. Matthews Ave, Urbana, IL 61801, USA; Carl R. Woese Institute for Genomic Biology, University of Illinois Urbana-Champaign, 1206 W. Gregory Drive, Urbana, IL 61801, USA; Institute for Molecular Bio Science, Goethe University Frankfurt, Max-von-Laue Strasse 9, 60438 Frankfurt am Main, Germany; LOEWE Center for Translational Biodiversity Genomics (TBG), Senckenberganlage 25, 60325 Frankfurt am Main, Germany; Department of Chemistry, University of Manitoba, 66 Chancellors Circle, Winnipeg, MB R3T 2N2, Canada; Biochemistry of Microorganisms, Faculty of Life Sciences: Food, Nutrition, and Health, University of Bayreuth, Fritz-Hornschuch-Straße 13, 95326 Kulmbach, Germany; Université Paris Cité - Inserm Unit 1284, 75015 Paris, France; Translational Genome Mining for Natural Products, Generare Bioscience, 75011 Paris, Île-de-France, France; Newcastle University, Biosciences Institute, Catherine Cookson Building, Newcastle upon Tyne, NE2 4HH, UK; SIB Swiss Institute of Bioinformatics, Centre Medical Universitaire, 1 rue Michel Servet, 1211 Geneva 4, Switzerland; Department of Microbiology, University of Helsinki, Viikinkaari 9, 00017, Helsinki, Finland; Manchester Institute of Biotechnology, Department of Chemistry, School of Natural Sciences, Faculty of Science and Engineering, University of Manchester, 131 Princess Street, Manchester M1 7DN, UK; Research Center for Food Technology and Processing, Research Organization of Agriculture and Food, National Research and Innovation Agency (BRIN), Jl. Jogja - Wonosari Km 31.5, DI. Yogyakarta 55861, Indonesia; Microbial Metabolites for Food Research Group, National Research and Innovation Agency (BRIN), Jl. Jogja - Wonosari Km 31.5, Gunungkidul, DI. Yogyakarta 55861, Indonesia; Microbial and Environmental Genomics Group, J. Craig Venter Institute, 4120 Capricorn Lane, La Jolla, CA 92037, USA; Translational Genome Mining for Natural Products, Interfaculty Institute of Microbiology and Infection Medicine Tübingen (IMIT), Interfaculty Institute for Biomedical Informatics (IBMI), University of Tübingen, Sand 14, 72076 Tübingen, Germany; NAICONS Srl, viale Ortles 22/4, 20139 Milan, Italy; Department of Chemistry, Life Sciences and Environmental Sustainability, University of Parma, Biosciences complex – Pavilion 02, Sciences and Technology Campus 11/A, 43124 Parma, Italy; Institute for Organic Chemistry, Leibniz Universität Hannover, Schneiderberg 38, 30167 Hannover, Germany; VIB-KU Leuven Center for Microbiology, Flanders Institute for Biotechnology, Kasteelpark Arenberg 31, 3001 Leuven, Belgium; Department of Biology, Laboratory for Biomolecular Discovery & Engineering, KU Leuven, Kasteelpark Arenberg 31, 3001 Leuven, Belgium; Department of Biosystems, Biosensors Group, KU Leuven, Willem de Croylaan 42, box 2428, 3001 Leuven, Belgium; Leibniz Institute DSMZ - German Collection of Microorganisms and Cell Cultures GmbH, Inhoffenstraße 7B, 38124Braunschweig, Germany; Industrial Genomics Laboratory, Centro de Biotecnología FEMSA, Escuela de Ingeniería y Ciencias, Tecnológico de Monterrey, Av. Eugenio Garza Sada 2501sur, Nuevo Leon, 64700, México; Center for Marine Biotechnology and Biomedicine, Scripps Institution of Oceanography, University of California San Diego, 9500 Gilman Drive, La Jolla, CA 92093-0212, USA; Department of Biosciences, Swansea University, Singleton Park, Swansea, SA2 8PP, UK; Industrial Genomics Laboratory, Centro de Biotecnología FEMSA, Escuela de Ingeniería y Ciencias, Tecnológico de Monterrey, Av. Eugenio Garza Sada 2501sur, Nuevo Leon, 64700, México; Department of Microbiology and Cell Science, University of Florida, 1355 Museum Drive, Gainesville, Florida, 32611, USA; University of Florida Genetics Institute, University of Florida, 2033 Mowry Rd, Gainesville, FL 32611, USA; Centro de Ciencias Matemáticas National Autonomous University of Mexico (UNAM) Antigua Carretera a Pátzcuaro # 8701, Sin Nombre, Residencial San José de la Huerta, 58089 Morelia, Mich; Helmholtz Institute for Pharmaceutical Research Saarland (HIPS), Helmholtz Centre for Infection Research (HZI), Campus E8.1, 66123 Saarbrücken, Germany; Saarland University, Campus E8.1, 66123 Saarbrücken, Germany; Biosphere Sciences and Engineering Division, Carnegie Institution for Science, 3520 San Martin Dr, Baltimore, MD, 21218, USA; Program in Chemical Biology, University of Michigan, 210 Washtenaw Avenue, Ann Arbor, MI, USA; Life Sciences Institute, University of Michigan, 210 Washtenaw Avenue, Ann Arbor, Michigan, USA; Department of Chemical and Pharmaceutical Biology, University of Groningen, Antonius Deusinglaan 1, 9713AV Groningen, The Netherlands; Department of Chemistry, The University of Hong Kong, Pokfulam Road, Hong Kong, China; IOCB Prague, Czech Academy of Science, Flemingovo náměstí 542/2, 160 00 Praha 6, Czech Republic; Department of Biomolecular Chemistry, Leibniz Institute for Natural Product Research and Infection Biology (HKI), Adolf-Reichwein-Straße 23, 07745 Jena, Germany; Institute of Infectious Disease Research, McMaster University, 1280 Main Street West, Hamilton, Ontario, L8S4L8, Canada; Department of Biology, McMaster University, 1280 Main Street West, Hamilton, Ontario, L8S4K1, Canada; Department of Pharmaceutical Sciences, University of Basel, Klingelbergstrasse 50, 4056 Basel, Switzerland; School of Molecular Sciences, University of Western Australia, 35 Stirling Highway, Perth 6009, Australia; Department of Biotechnology and Nanomedicine, SINTEF Industry, P.O.Box 4760 Torgard, N-7465 Trondheim, Norway; The Novo Nordisk Foundation Center for Biosustainability, Technical University of Denmark, Building 220, Søltofts Plads, 2800 Kongens Lyngby, Denmark; Bioinformatics Group, Wageningen University & Research, Droevendaalsesteeg 1, 6708 PB Wageningen, The Netherlands; Microbiology Group, Wageningen University & Research, Droevendaalsesteeg 1, 6708 PB Wageningen, The Netherlands; Corteva Agriscience, 9330 Zionsville Road, Indianapolis, Indiana 46268, USA; Natural Products Research Institute, College of Pharmacy, Seoul National University, 101 Daehak-ro, Jongro-gu, Seoul 110-744, Korea; Molecular Targets Program, Center for Cancer Research, National Cancer Institute, Frederick, Maryland 21702-1201, USA; Production Host Engineering Team, VTT Technical Research Centre of Finland Ltd, Maarintie 3, 02150 Espoo, Finland; Interfaculty Institute of Microbiology and Infection Medicine Tübingen, Microbial Bioactive Compounds, University of Tübingen, Auf der Morgenstelle 28, 72076, Tübingen, Germany; NAICONS Srl, viale Ortles 22/4, 20139 Milan, Italy; The Novo Nordisk Foundation Center for Biosustainability, Technical University of Denmark, Building 220, Søltofts Plads, 2800 Kongens Lyngby, Denmark; College of Pharmacy and Drug Information Research Institute, Sookmyung Women's University, 100 Cheongpa-ro 47 gil, Seoul 04310, Republic of Korea; Bioinformatics Group, Wageningen University & Research, Droevendaalsesteeg 1, 6708 PB Wageningen, The Netherlands; Natural Products Research Institute, College of Pharmacy, Seoul National University, 101 Daehak-ro, Jongro-gu, Seoul 110-744, Korea; Institute for Molecular Bio Science, Goethe University Frankfurt, Max-von-Laue Strasse 9, 60438 Frankfurt am Main, Germany; LOEWE Center for Translational Biodiversity Genomics (TBG), Senckenberganlage 25, 60325 Frankfurt am Main, Germany; Department of Chemistry and Biochemistry, Laboratories of Molecular Recognition, Florida State University, 95 Chieftan Way, Tallahassee, FL 32306, USA; College of Pharmacy and Drug Information Research Institute, Sookmyung Women's University, 100 Cheongpa-ro 47 gil, Seoul 04310, Republic of Korea; Natural Products Research Institute, College of Pharmacy, Seoul National University, 101 Daehak-ro, Jongro-gu, Seoul 110-744, Korea; Department of Applied Biology, College of Agriculture and Life Sciences, Chonnam National University, 77 Yongbong-ro, Buk-gu, Gwangju, 61186, South Korea; Research Institute of Cell Culture, Yeungnam University, 280 Daehak-ro Gyeongsan-Si, Gyeongsangbuk-do 38541, Republic of Korea; Department of Pharmacology, College of Medicine, Dongguk University, Dongdae-ro 123, Gyeongju-si, Gyeongsangbuk-do 38066, Republic of Korea; College of Pharmacy and Integrated Research Institute for Drug Development, Dongguk University-Seoul, Dongguk-ro 32, Goyang 10326, Republic of Korea; Department of Chemical and Biomolecular Engineering, Korea Advanced Institute of Science and Technology (KAIST), Daejeon 34141, Republic of Korea; Department of Paleobiotechnology, Leibniz Institute for Natural Product Research and Infection Biology Hans Knöll Institute, Beutenbergstr. 11a, 07745 Jena, Germany; Bioinformatics Group, Wageningen University & Research, Droevendaalsesteeg 1, 6708 PB Wageningen, The Netherlands; Chair of Technical Biochemistry, Technical University of Dresden, Bergstraße 66, 01069 Dresden, Germany; Institute of Biology, Leiden University, Sylviusweg 72, 2333BE Leiden, The Netherlands; DTU Bioengineering, Technical University of Denmark, 2800 Kgs. Lyngby, Denmark; Helmholtz Institute for Pharmaceutical Research Saarland (HIPS), Helmholtz Centre for Infection Research (HZI), Campus E8.1, 66123 Saarbrücken, Germany; Chair of Technical Biochemistry, Technical University of Dresden, Bergstraße 66, 01069 Dresden, Germany; Translational Genome Mining for Natural Products, Interfaculty Institute of Microbiology and Infection Medicine Tübingen (IMIT), Interfaculty Institute for Biomedical Informatics (IBMI), University of Tübingen, Sand 14, 72076 Tübingen, Germany; Department of Chemical and Biomolecular Engineering, University of Delaware, Newark, DE, USA; Helmholtz Institute for Pharmaceutical Research Saarland (HIPS), Helmholtz Centre for Infection Research (HZI), Campus E8.1, 66123 Saarbrücken, Germany; Saarland University, Campus E8.1, 66123 Saarbrücken, Germany; Innovative Genomics Institute, University of California Berkeley, 2151Berkeley Way, Berkeley, CA 94720, USA; Department of Earth and Planetary Science, University of California, 307 McCone Hall Berkeley, CA 94720-4767, USA; Helmholtz Institute for Pharmaceutical Research Saarland (HIPS), Helmholtz Centre for Infection Research (HZI), Campus E8.1, 66123 Saarbrücken, Germany; Saarland University, Campus E8.1, 66123 Saarbrücken, Germany; German Centre for Infection Research (DZIF), Inhoffenstr. 7, 38124 Hannover-Braunschweig, Germany; Center of Infectious Diseases, Leiden University Medical Center, Albinusdreef 2, 2333 ZA Leiden, Netherlands; The Novo Nordisk Foundation Center for Biosustainability, Technical University of Denmark, Building 220, Søltofts Plads, 2800 Kongens Lyngby, Denmark; Bioinformatics department, Endogenomiks, El Marqués, Querétaro, Mexico; Université Paris Cité - Inserm Unit 1284, 75015 Paris, France; Department of Bioscience Engineering, Research Group Laboratory of Applied Microbiology and Biotechnology, University of Antwerp, Groenenborgerlaan 171, 2020 Antwerp, Belgium; U-MaMi Centre of Excellence, Middelheimlaan 1, 2020 Antwerp, Belgium; Department of Chemical and Biomolecular Engineering, Korea Advanced Institute of Science and Technology (KAIST), Daejeon 34141, Republic of Korea; College of Pharmacy and Drug Information Research Institute, Sookmyung Women's University, 100 Cheongpa-ro 47 gil, Seoul 04310, Republic of Korea; Program in Chemical Biology, University of Michigan, 210 Washtenaw Avenue, Ann Arbor, MI, USA; Life Sciences Institute, University of Michigan, 210 Washtenaw Avenue, Ann Arbor, Michigan, USA; Institute of Pharmaceutical Biology and Biotechnology, University of Marburg, Robert-Koch-Str. 4, 35037 Marburg, Germany; Department of Chemistry, The University of Hong Kong, Pokfulam Road, Hong Kong, China; Industrial Genomics Laboratory, Centro de Biotecnología FEMSA, Escuela de Ingeniería y Ciencias, Tecnológico de Monterrey, Av. Eugenio Garza Sada 2501sur, Nuevo Leon, 64700, México; Integrative Biology Research Unit, The Institute for Obesity Research, Tecnológico de Monterrey, Av. Eugenio Garza Sada 2501 sur, Nuevo Leon, 64700. México; Bioinformatics Group, Wageningen University & Research, Droevendaalsesteeg 1, 6708 PB Wageningen, The Netherlands; Department of Chemistry, The University of Hong Kong, Pokfulam Road, Hong Kong, China; School of Innovation and Sustainability, De La Salle University, Laguna Boulevard, LTI Spine Road, Brgys. Biñan and Malamig, 4024 Biñan City, Laguna, Philippines; Department of Chemistry, De La Salle University, 2401 Taft Avenue, 0922 Manila, Philippines; Institute of Biology, Leiden University, Sylviusweg 72, 2333BE Leiden, The Netherlands; University of Strathclyde, Strathclyde Institute of Pharmacy and Biomedical Sciences, Glasgow, G4 0RE UK; Department of Pharmaceutical Sciences, Oregon State University, 1601 SW Jefferson Way, Corvallis, OR 97331-3507, USA; Department of Biotechnology and Biomedicine, Technical University of Denmark, Søltofts Plads, Building 221, 2800 Kgs. Lyngby, Denmark; Industrial Genomics Laboratory, Centro de Biotecnología FEMSA, Escuela de Ingeniería y Ciencias, Tecnológico de Monterrey, Av. Eugenio Garza Sada 2501sur, Nuevo Leon, 64700, México; Leibniz Institute DSMZ - German Collection of Microorganisms and Cell Cultures GmbH, Inhoffenstraße 7B, 38124Braunschweig, Germany; Technical University of Braunschweig, Institute of Microbiology, Rebenring 56, 38106 Braunschweig, Germany; Department of Organic Chemistry, Institute of Chemistry, University of Campinas (UNICAMP), Rua Monteiro Lobato 270, Campinas, São Paulo, 13.083-862, Brazil; Program in Chemical Biology, University of Michigan, 210 Washtenaw Avenue, Ann Arbor, MI, USA; Life Sciences Institute, University of Michigan, 210 Washtenaw Avenue, Ann Arbor, Michigan, USA; Ferrier Research Institute, Victoria University of Wellington, Kelburn Parade, Wellington 6012, New Zealand; Maurice Wilkins Centre for Molecular Biodiscovery, Victoria University of Wellington, Kelburn Parade, Wellington 6012, New Zealand; Fungal Natural Products, Westerdijk Fungal Biodiversity Institute, Uppsalalaan 8, 3584CT Utrecht, The Netherlands; Bioinformatics Group, Wageningen University & Research, Droevendaalsesteeg 1, 6708 PB Wageningen, The Netherlands; Theoretical Biology and Bioinformatics, Department of Biology, Faculty of Science, Utrecht University, 3584 CH Utrecht, The Netherlands; Department of Biological and Chemical Engineering, Aarhus University, Gustav Wieds Vej 10, 8000 Aarhus C, Denmark; Department of Life Technologies, University of Turku, Tykistökatu 6, FIN-20520 Turku, Finland; Department of Chemistry, University of Illinois Urbana-Champaign, 600 S. Matthews Ave, Urbana, IL 61801, USA; Helmholtz Institute for Pharmaceutical Research Saarland (HIPS), Helmholtz Centre for Infection Research (HZI), Campus E8.1, 66123 Saarbrücken, Germany; Saarland University, Campus E8.1, 66123 Saarbrücken, Germany; German Centre for Infection Research (DZIF), Inhoffenstr. 7, 38124 Hannover-Braunschweig, Germany; Department of Biotechnology and Nanomedicine, SINTEF Industry, P.O.Box 4760 Torgard, N-7465 Trondheim, Norway; School of Molecular Sciences, University of Western Australia, 35 Stirling Highway, Perth 6009, Australia; Institute of Pharmacy, Freie Universität Berlin, Königin-Luise-Str. 2+4, 14195 Berlin, Germany; Instituto de Pesquisas de Produtos Naturais Walter Mors, Universidade Federal do Rio de Janeiro, CCS - Av. Carlos Chagas Filho, 373 - Bloco H - Cidade Universitária, Rio de Janeiro - RJ, 21941-599, Brazil; Department of Crop Protection, Instituto de Hortofruticultura Subtropical y Mediterránea “La Mayora” (IHSM-UMA-CSIC), Campus Universitario de Teatinos, 29010, Málaga, Spain; Department of Microbiology, Faculty of Science, Campus Universitario de Teatinos s/n, University of Málaga, 29010 Málaga, Spain; Department of Genetics and Biotechnology, Ivan Franko National University of Lviv, Hrushevskoho st 4, Lviv 79005, Ukraine; German-Ukrainian Core of Excellence in Natural Products Research, Zelena str. 20, Lviv 79005, Ukraine; US Department of Energy Joint Genome Institute, Lawrence Berkeley National Laboratory, 1 Cyclotron Road, Berkeley, CA 94720, USA; Environmental Genomics and Systems Biology Division, Lawrence Berkeley National Laboratory, 1 Cyclotron Road, Berkeley, CA 94720, USA; Institute of Pharmaceutical Biology, University of Bonn, Nussallee 6, 53115 Bonn, Germany; Computational Biology Lab, National Agri-Food and Biomanufacturing Institute (NABI), Sector 81, S.A.S. Nagar, Mohali, Punjab 140306, India; Institute of Biology, Leiden University, Sylviusweg 72, 2333BE Leiden, The Netherlands; Department of Crop Protection, Instituto de Hortofruticultura Subtropical y Mediterránea “La Mayora” (IHSM-UMA-CSIC), Campus Universitario de Teatinos, 29010, Málaga, Spain; Department of Microbial Ecology, Netherlands Institute of Ecology (NIOO-KNAW), Droevendaalsesteeg 10 6708 PB Wageningen, The Netherlands; Computational Biology Lab, National Agri-Food and Biomanufacturing Institute (NABI), Sector 81, S.A.S. Nagar, Mohali, Punjab 140306, India; Regional Centre for Biotechnology, NCR Biotech Science Cluster, 3rd Milestone, Faridabad-Gurugram Expressway, Faridabad - 121001 Haryana (NCR Delhi), India; Helmholtz Institute for Pharmaceutical Research Saarland (HIPS), Helmholtz Centre for Infection Research (HZI), Campus E8.1, 66123 Saarbrücken, Germany; Natural Products Research Institute, College of Pharmacy, Seoul National University, 101 Daehak-ro, Jongro-gu, Seoul 110-744, Korea; Centro de Investigaciones en Productos Naturales (CIPRONA), Universidad de Costa Rica, San José, 11501-2060, Costa Rica; Centro Nacional de Innovaciones Biotecnológicas (CENIBiot), CeNAT-CONARE, 1174-1200, San José, Costa Rica; Instituto de Investigaciones Farmacéuticas (INIFAR), Facultad de Farmacia, Universidad de Costa Rica, San José, 11501-2060, Costa Rica; Department of Crop Protection, Instituto de Hortofruticultura Subtropical y Mediterránea “La Mayora” (IHSM-UMA-CSIC), Campus Universitario de Teatinos, 29010, Málaga, Spain; Department of Microbiology, Faculty of Science, Campus Universitario de Teatinos s/n, University of Málaga, 29010 Málaga, Spain; College of Pharmacy and Drug Information Research Institute, Sookmyung Women's University, 100 Cheongpa-ro 47 gil, Seoul 04310, Republic of Korea; Department of Microbiology and Immunology at the Doherty Institute, University of Melbourne, Grattan Street, Parkville Victoria, Victoria 3000, Australia; Institute of Microbiology, Eidgenössische Technische Hochschule (ETH) Zurich, Vladimir-Prelog Weg 4, 8093 Zurich, Switzerland; Translational Genome Mining for Natural Products, Interfaculty Institute of Microbiology and Infection Medicine Tübingen (IMIT), Interfaculty Institute for Biomedical Informatics (IBMI), University of Tübingen, Sand 14, 72076 Tübingen, Germany; Department of Pharmaceutical Sciences, University of Basel, Klingelbergstrasse 50, 4056 Basel, Switzerland; Department of Plant Pathology, University of California Davis, One Shields Avenue, Davis, CA 95616-8751, USA; Department of Chemistry, Aarhus University, Langelandsgade 140, DK-8000, Aarhus C, Denmark; Interdisciplinary Centre of Marine and Environmental Research (CIIMAR/CIMAR), University of Porto, 4450-208 Matosinhos, Portugal; Institute of Pharmaceutical Biology and Biotechnology, University of Marburg, Robert-Koch-Str. 4, 35037 Marburg, Germany; Department of Chemistry, University of Illinois Urbana-Champaign, 600 S. Matthews Ave, Urbana, IL 61801, USA; Institute of Biology, Leiden University, Sylviusweg 72, 2333BE Leiden, The Netherlands; The Novo Nordisk Foundation Center for Biosustainability, Technical University of Denmark, Building 220, Søltofts Plads, 2800 Kongens Lyngby, Denmark; Department of Biotechnology and Biomedicine, Technical University of Denmark, Søltofts Plads, Building 221, 2800 Kgs. Lyngby, Denmark; Institute of Biology, Leiden University, Sylviusweg 72, 2333BE Leiden, The Netherlands; Unidad de Genómica Avanzada, Centro de Investigación y de Estudios Avanzados del Instituto Politécnico Nacional, Km 9.6 Libramiento Norte Carretera Irapuato-León, 36824, Irapuato, Mexico; Bioinformatics department, Endogenomiks, El Marqués, Querétaro, Mexico; Institute for Molecular Systems Biology, ETH Zürich, Otto-Stern-Weg 3, 8093 Zürich, Switzerland; Center for Marine Biotechnology and Biomedicine, Scripps Institution of Oceanography, University of California San Diego, 9500 Gilman Drive, La Jolla, CA 92093-0212, USA; Department of Chemistry, University of Manitoba, 66 Chancellors Circle, Winnipeg, MB R3T 2N2, Canada; Department of Microbiology and Cell Science, University of Florida, 1355 Museum Drive, Gainesville, Florida, 32611, USA; Interfaculty Institute of Microbiology and Infection Medicine Tübingen, Microbial Bioactive Compounds, University of Tübingen, Auf der Morgenstelle 28, 72076, Tübingen, Germany; Laboratorio de Microbiología Molecular y Biotecnología Ambiental, Centro de Biotecnología DAL, Universidad Técnica Federico Santa María, Avenida España 1680, 2390123, Valparaíso, Chile; Corteva Agriscience, 9330 Zionsville Road, Indianapolis, Indiana 46268, USA; School of Molecular Sciences, University of Western Australia, 35 Stirling Highway, Perth 6009, Australia; Department of Chemistry and BioDiscovery Institute, University of North Texas, 1155 Union Circle, Denton, Texas 76203, USA; Helmholtz Institute for Pharmaceutical Research Saarland (HIPS), Helmholtz Centre for Infection Research (HZI), Campus E8.1, 66123 Saarbrücken, Germany; German Centre for Infection Research (DZIF), Inhoffenstr. 7, 38124 Hannover-Braunschweig, Germany; Helmholtz Centre for Infection Research (HZI), Inhoffenstr. 7, 38124 Braunschweig, Germany; Interfaculty Institute of Microbiology and Infection Medicine Tübingen, Microbial Bioactive Compounds, University of Tübingen, Auf der Morgenstelle 28, 72076, Tübingen, Germany; Masaryk University, Faculty of Science, RECETOX, Kamenice 753/5, 625 00 Brno, Czech Republic; Centro de Ciencias Matemáticas National Autonomous University of Mexico (UNAM) Antigua Carretera a Pátzcuaro # 8701, Sin Nombre, Residencial San José de la Huerta, 58089 Morelia, Mich; School of Biological Sciences, University of Bristol, Life Sciences Building, 24 Tyndall Avenue, Bristol, BS8 1TQ, UK; School of Chemistry, University of Bristol, Cantock's Close, Bristol, BS8 1TS, UK; Fungal Natural Products, Westerdijk Fungal Biodiversity Institute, Uppsalalaan 8, 3584CT Utrecht, The Netherlands; Institute of Biotechnology, Helsinki Institute of Life Science, University of Helsinki, Viikinkaari 5, 00790, Helsinki, Finland; Department of Chemistry, University of Zurich,Winterthurerstrasse 190, 8057 Zurich, Switzerland; College of Pharmacy, Yeungnam University, 280 Daehak-ro Gyeongsan-Si, Gyeongsangbuk-do 38541, Republic of Korea; Research Institute of Cell Culture, Yeungnam University, 280 Daehak-ro Gyeongsan-Si, Gyeongsangbuk-do 38541, Republic of Korea; Department of Biology, University of Padova, Via U. Bassi, 58/B, 35121 Padova, Italy; Botanical Garden, University of Padova, Via Orto Botanico 15, 35123 Padova, Italy; Computational Biology Lab, National Agri-Food and Biomanufacturing Institute (NABI), Sector 81, S.A.S. Nagar, Mohali, Punjab 140306, India; Department of Chemical and Pharmaceutical Biology, University of Groningen, Antonius Deusinglaan 1, 9713AV Groningen, The Netherlands; Department of Biosciences, Swansea University, Singleton Park, Swansea, SA2 8PP, UK; NAICONS Srl, viale Ortles 22/4, 20139 Milan, Italy; Department of Pharmaceutical Sciences, University of Basel, Klingelbergstrasse 50, 4056 Basel, Switzerland; Department of Biological Sciences and Evolutionary Studies Initiative, Vanderbilt University, 465 21st Ave S, Nashville, TN 37232, USA; Interfaculty Institute of Microbiology and Infection Medicine Tübingen, Microbial Bioactive Compounds, University of Tübingen, Auf der Morgenstelle 28, 72076, Tübingen, Germany; German Centre for Infection Research (DZIF), Partner Site Tübingen, Hoppe-Seyler-Straße 3, 72076 Tübingen, Germany; Institute of Microbiology, Eidgenössische Technische Hochschule (ETH) Zurich, Vladimir-Prelog Weg 4, 8093 Zurich, Switzerland; Interfaculty Institute of Microbiology and Infection Medicine Tübingen, Microbial Bioactive Compounds, University of Tübingen, Auf der Morgenstelle 28, 72076, Tübingen, Germany; Department of Microbial Drugs, Helmholtz Centre for Infection Research (HZI), Inhoffenstr. 7, 38124 Braunschweig, Germany; Institute of Microbiology, Technische Universität Braunschweig, Spielmannstraße 7, 38106 Braunschweig, Germany; Department of Biological and Chemical Engineering, Aarhus University, Gustav Wieds Vej 10, 8000 Aarhus C, Denmark; Center for Marine Biotechnology and Biomedicine, Scripps Institution of Oceanography, University of California San Diego, 9500 Gilman Drive, La Jolla, CA 92093-0212, USA; The Novo Nordisk Foundation Center for Biosustainability, Technical University of Denmark, Building 220, Søltofts Plads, 2800 Kongens Lyngby, Denmark; Helmholtz Institute for Pharmaceutical Research Saarland (HIPS), Helmholtz Centre for Infection Research (HZI), Campus E8.1, 66123 Saarbrücken, Germany; Saarland University, Campus E8.1, 66123 Saarbrücken, Germany; Institute for Molecular Bio Science, Goethe University Frankfurt, Max-von-Laue Strasse 9, 60438 Frankfurt am Main, Germany; LOEWE Center for Translational Biodiversity Genomics (TBG), Senckenberganlage 25, 60325 Frankfurt am Main, Germany; Department of Biosciences, Swansea University, Singleton Park, Swansea, SA2 8PP, UK; Bioinformatics Group, Wageningen University & Research, Droevendaalsesteeg 1, 6708 PB Wageningen, The Netherlands; DE SANGOSSE, Bonnel, 47480, Pont-Du-Casse, France; Laboratoire de Recherche en Sciences Végétales, Université de Toulouse, CNRS, Université Toulouse III, Toulouse INP, 24 Chemin de Borde Rouge, Auzeville, 31320, Auzeville-Tolosane, France; Institute of Biology, Leiden University, Sylviusweg 72, 2333BE Leiden, The Netherlands; Department of Biology, Laboratory for Biomolecular Discovery & Engineering, KU Leuven, Kasteelpark Arenberg 31, 3001 Leuven, Belgium; Bioinformatics Group, Wageningen University & Research, Droevendaalsesteeg 1, 6708 PB Wageningen, The Netherlands; Department of Biological and Chemical Engineering, Aarhus University, Gustav Wieds Vej 10, 8000 Aarhus C, Denmark; Institute for Microbial Biotechnology and Metagenomics, Department of Biotechnology, University of the Western Cape, Robert Sobukwe Rd, Bellville, 7535, South Africa; Department of Molecular Microbiology, John Innes Centre, Norwich Research Park, Norwich, NR4 7UH, United Kingdome, Norwich, UK; Department of Biological and Chemical Engineering, Aarhus University, Gustav Wieds Vej 10, 8000 Aarhus C, Denmark; US Department of Energy Joint Genome Institute, Lawrence Berkeley National Laboratory, 1 Cyclotron Road, Berkeley, CA 94720, USA; Institute of Pharmaceutical Biology, University of Bonn, Nussallee 6, 53115 Bonn, Germany; The Novo Nordisk Foundation Center for Biosustainability, Technical University of Denmark, Building 220, Søltofts Plads, 2800 Kongens Lyngby, Denmark; Institute of Biology, Leiden University, Sylviusweg 72, 2333BE Leiden, The Netherlands; Department of Microbial Ecology, Netherlands Institute of Ecology (NIOO-KNAW), Droevendaalsesteeg 10 6708 PB Wageningen, The Netherlands; Manchester Institute of Biotechnology, Department of Chemistry, School of Natural Sciences, Faculty of Science and Engineering, University of Manchester, 131 Princess Street, Manchester M1 7DN, UK; Department of Chemistry, University of Manitoba, 66 Chancellors Circle, Winnipeg, MB R3T 2N2, Canada; Department of Pharmaceutical Sciences, University of Basel, Klingelbergstrasse 50, 4056 Basel, Switzerland; VIB-KU Leuven Center for Microbiology, Flanders Institute for Biotechnology, Kasteelpark Arenberg 31, 3001 Leuven, Belgium; The Rosalind Franklin Institute, R113 Rutherford Appleton Laboratory, Harwell Science and Innovation Campus, Didcot, Oxfordshire, OX11 0QX, UK; School of Biological Sciences, University of Bristol, Life Sciences Building, 24 Tyndall Avenue, Bristol, BS8 1TQ, UK; School of Applied Sciences, University of the West of England, Frenchay Campus, Coldharbour Lane, Bristol, BS16 1QY, UK; The Novo Nordisk Foundation Center for Biosustainability, Technical University of Denmark, Building 220, Søltofts Plads, 2800 Kongens Lyngby, Denmark; Department of Chemistry and Biomolecular Sciences, University of Ottawa, 10 Marie Curie Private, Ottawa, Ontario, K1N 6N5, Canada; Center for Marine Biotechnology and Biomedicine, Scripps Institution of Oceanography, University of California San Diego, 9500 Gilman Drive, La Jolla, CA 92093-0212, USA; Microbial and Environmental Genomics Group, J. Craig Venter Institute, 4120 Capricorn Lane, La Jolla, CA 92037, USA; Department of Life Technologies, University of Turku, Tykistökatu 6, FIN-20520 Turku, Finland; Natural Products Discovery Center, The Herbert Wertheim UF Scripps Institute for Biomedical Innovation & Technology, University of Florida, Jupiter, Florida 33458, USA; Synthetic Biology and Enzyme Engineering Laboratory, Department of Chemical and Biological Engineering, Korea University, Anam-ro 145, Seongbuk-gu, Seoul 02841, Republic of Korea; Institute of Plant and Food Science, Department of Biology, School of Life Sciences, Southern University of Science and Technology, 1088 Xueyuan Avenue, Shenzhen 518055, P.R. China; College of Pharmaceutical Science & Collaborative Innovation Center of Yangtze River Delta Region Green Pharmaceuticals, Zhejiang University of Technology, Chaowang Raod 18, Hangzhou 310014, China; Translational Genome Mining for Natural Products, Interfaculty Institute of Microbiology and Infection Medicine Tübingen (IMIT), Interfaculty Institute for Biomedical Informatics (IBMI), University of Tübingen, Sand 14, 72076 Tübingen, Germany; Institute of Pharmaceutical Biology, University of Bonn, Nussallee 6, 53115 Bonn, Germany; Leibniz Institute DSMZ - German Collection of Microorganisms and Cell Cultures GmbH, Inhoffenstraße 7B, 38124Braunschweig, Germany; Institute of Chemical, Environmental and Bioscience Engineering, TU Wien, Gumpendorfer Strasse 1a, 1060 Vienna, Austria; Bioinformatics Group, Wageningen University & Research, Droevendaalsesteeg 1, 6708 PB Wageningen, The Netherlands; Department of Biochemistry, University of Johannesburg, C2 Lab Building 224, Kingsway Campus, Cnr University & Kingsway Road, Auckland Park, Johannesburg 2006, South Africa; Department of Chemistry, Simon Fraser University, 8888 University Drive, Burnaby, British Columbia, V5A 1S6, Canada; The Novo Nordisk Foundation Center for Biosustainability, Technical University of Denmark, Building 220, Søltofts Plads, 2800 Kongens Lyngby, Denmark; Bioinformatics Group, Wageningen University & Research, Droevendaalsesteeg 1, 6708 PB Wageningen, The Netherlands

## Abstract

Specialized or secondary metabolites are small molecules of biological origin, often showing potent biological activities with applications in agriculture, engineering and medicine. Usually, the biosynthesis of these natural products is governed by sets of co-regulated and physically clustered genes known as biosynthetic gene clusters (BGCs). To share information about BGCs in a standardized and machine-readable way, the Minimum Information about a Biosynthetic Gene cluster (MIBiG) data standard and repository was initiated in 2015. Since its conception, MIBiG has been regularly updated to expand data coverage and remain up to date with innovations in natural product research. Here, we describe MIBiG version 4.0, an extensive update to the data repository and the underlying data standard. In a massive community annotation effort, 267 contributors performed 8304 edits, creating 557 new entries and modifying 590 existing entries, resulting in a new total of 3059 curated entries in MIBiG. Particular attention was paid to ensuring high data quality, with automated data validation using a newly developed custom submission portal prototype, paired with a novel peer-reviewing model. MIBiG 4.0 also takes steps towards a rolling release model and a broader involvement of the scientific community. MIBiG 4.0 is accessible online at https://mibig.secondarymetabolites.org/.

## Graphical Abstract

**Graphical Abstract:**
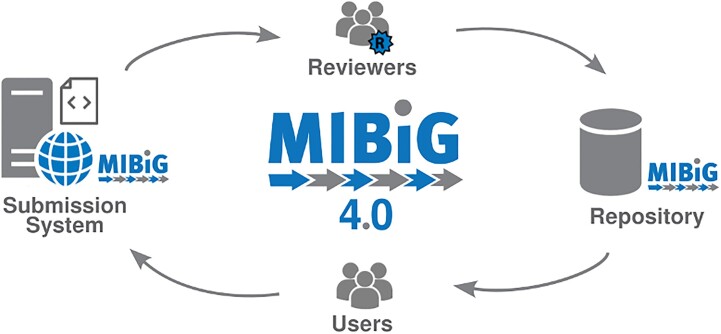

## Introduction

Many organisms are prolific producers of small molecules known as specialized or secondary metabolites (SMs). These molecules often show a diversity of potent biological activities, which have been leveraged for the development of numerous drugs (1,2). SMs are generally hypothesized to increase the fitness of the producing organism or its host. In microbes, the biosynthetic genes required for the production of an SM are co-regulated and frequently physically clustered in the genome, in a so-called biosynthetic gene cluster (BGC), and often transferred horizontally (3). BGCs, which by definition consist of two or more genes, encode the proteins/enzymes used in biosynthesis, resistance and regulation of SMs and are the object of ‘genome mining’ strategies that leverage analysis of genome sequence data for the discovery of (novel) metabolites (4).

Over the last decades, various methods using manually curated detection rules based on prior knowledge (5–7), and more recently, machine learning-based tools for genome mining have been developed (8–12). These tools rely on accurately curated and machine-readable experimental data for annotation, rule definition and training purposes. Unfortunately, machine-readable data are neither readily available from the scientific literature nor universally required by journals to be directly deposited in databases. While there are efforts to mine data from the literature using computational methods (13,14), these approaches currently often come with limitations when compared with human curators and may not be compatible with copyright laws. Therefore, manual data curation performed by researchers remains the gold standard for the generation of machine-readable data.

The largest manually curated resource on SM BGCs is the Minimum Information about a Biosynthetic Gene Cluster (MIBiG) data repository (15). Initiated in 2015 and based on the MIBiG Data Standard, it now holds over 2500 hand-curated entries of experimentally validated BGCs and their products, alongside additional information such as biological activities and gene annotations. In rare cases, a single gene may be responsible for the biosynthesis of a natural product, such as a large non-ribosomal peptide synthases; these standalone genes are also entered into MIBiG due to their relevance to specialised metabolism. Conceptualized as an open data repository curated by and for the SM community, the MIBiG repository has seen three iterations of online community-driven data annotation and curation hackathons (also known as ‘annotathons’), with >250 participants from 33 countries (16,17). Despite its size, the MIBiG repository still only describes a part of the continuously growing known biosynthetic space, which motivates further efforts in curating and systemizing information on BGCs.

Here, we present version 4.0 of the MIBiG data standard and repository. Besides a thorough update of the underlying MIBiG data standard, we have substantially grown the number of available entries by initiating a large-scale community curation effort. In the first half of 2024, 267 contributors created 557 new entries and modified 590 existing entries in the scope of eight community annotathons (six general open events and two final data curation sessions with a more dedicated team). In this version of MIBiG, we focused on maintaining and further improving data quality in terms of completeness and accuracy. We encouraged contributors to fully complete entries before submission, which has significantly decreased the number of so-called minimum entries (entries with only the minimally required information) in the database. We also introduced a new peer-review model where modifications to entries are examined and approved by one or more volunteer expert reviewers, who can request corrections from data submitters. Additionally, we have established an initial prototype for efficient and standardized data submission, and during the annotathons we utilized a web interface (MIBiG Submission Portal) that allows for parallel, distributed data input featuring automated input validation. The latter refers to the tests that are performed by the submission portal itself to ensure the correct data types and formats are filled in. Together, these efforts further consolidate MIBiG as the leading database on experimentally characterized BGCs and prepare for the transition to a dynamic, rolling-release curation model.

## Materials and methods

### Rework of the MIBiG Data Standard

The MIBiG Data Standard (from here onwards, Data Standard) is the ‘blueprint’ of all allowed data in the MIBiG repository. It defines mandatory and optional data fields, allows the use of controlled vocabularies and automated validation and enables the organization of complex data in a consistent, human- and machine-readable way. In this update, we extensively revised the Data Standard to accommodate advances in SM research and to extend the scope and ease of (re-)use of covered (meta)data.

#### Literature references and evidence qualifiers

Previously, all literature references associated with a MIBiG entry were collected in a single block, making it difficult to locate the origin of specific experimental data. In this update, we reorganized the Data Standard such that each data category (e.g. biosynthetic information, compound details, etc.) has its own list of literature references. Furthermore, evidence qualifiers can be selected from a controlled vocabulary (e.g. ‘heterologous expression’) that concisely summarizes the experimental support for the claims. While newly added entries adhere to these changes, entries added in previous versions of MIBiG still follow the legacy format, and will be updated gradually over time. To summarize the data quality of an entry concisely, we also introduced a ‘Quality’ identifier, and it is possible to filter entries based on high, medium or questionable quality of data. Note that this label only reflects the presumed data quality of an MIBiG entry and does not address the quality of the underlying literature.

#### Biosynthesis information, multiple loci and class updates

Biosynthetic information is now organized in a ‘biosynthesis’ section, tracking biosynthetic types, modules, operons and newly introduced ‘biosynthetic path’, which allows contributors to describe cases where a single BGC can lead to multiple products or describe sub-clusters of genes that produce building blocks. The ‘multiple loci’ system has been re-introduced, allowing the specification of satellite genes or gene clusters that are involved in the biosynthesis but are not clustered with the ‘main’ BGC. Nevertheless, we still require that multiple biosynthetic genes are clustered in the same genomic region, to exclude non-clustered pathways. Furthermore, it is now possible to mark genes that are located within the boundaries of a BGC but do not partake in the biosynthesis, such as pseudo-genes or transposable elements. Additionally, we have separated biosynthetic classification from compound classification (e.g. we removed ‘alkaloid’ as a biosynthetic class) and introduced a custom biosynthesis-inspired chemical ontology for SMs (Supplementary Data 1, section 3.4) based on the work by Dewick (1). Furthermore, we have newly defined the non-ribosomal peptide synthetase Type VI (modular, non-condensation-domain peptide-bond-forming), extending the current classification (18).

#### Biological activity and resource integration

MIBiG also accepts additional BGC-related data. In this update, we have reworked fields registering the biological activity of BGC-associated SMs: activities are now considered properties of a specific assay, and a controlled vocabulary (Supplementary Data 1, section 3.3) is available for defining bioactivity in a reproducible way. Additionally, we have included an optional ‘Concentration’ field, allowing submission of both qualitative and quantitative bioactivity data. At the same time, additional metadata parameters increase the scope of the already extensive Data Standard, and as such MIBiG references external resources where possible. Newly introduced links include references to the Minimum Information about a Tailoring Enzyme (MITE) data repository for annotation of tailoring enzyme-encoding genes (19), and CyanoMetDB for compound information on cyanobacterial SMs (20).

### Community mobilization and data curation

**Figure 1.**
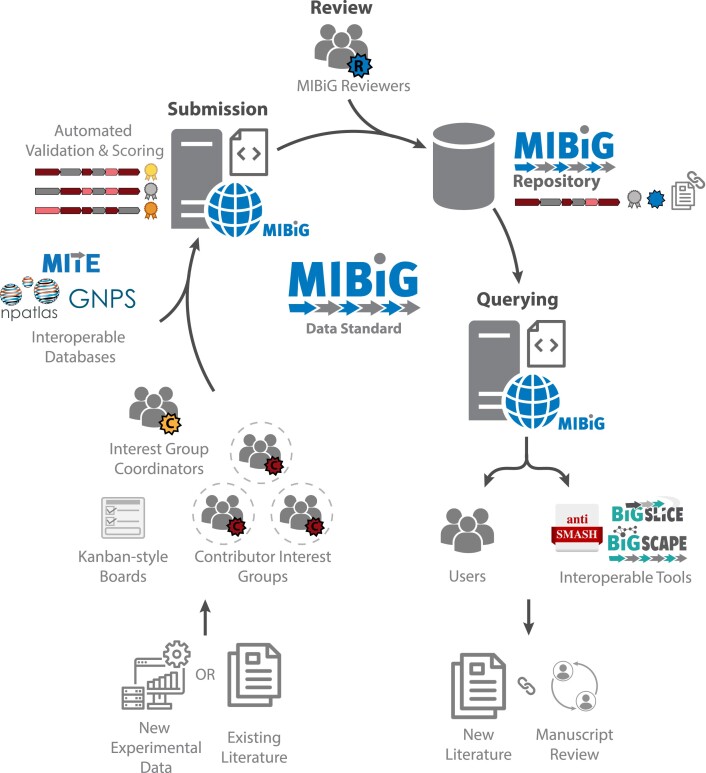
General workflow of the MIBiG annotation process. Data are submitted by annotathon contributors (organized by expertise into Interest Groups) or independent submitters to the database from new experimental data or existing/recent literature. The entries are then assessed by reviewers and revised when needed. Finally, they end up in the online MIBiG repository and become accessible by querying them online on the MIBiG web page or via interoperable tools.

**Figure 2.**
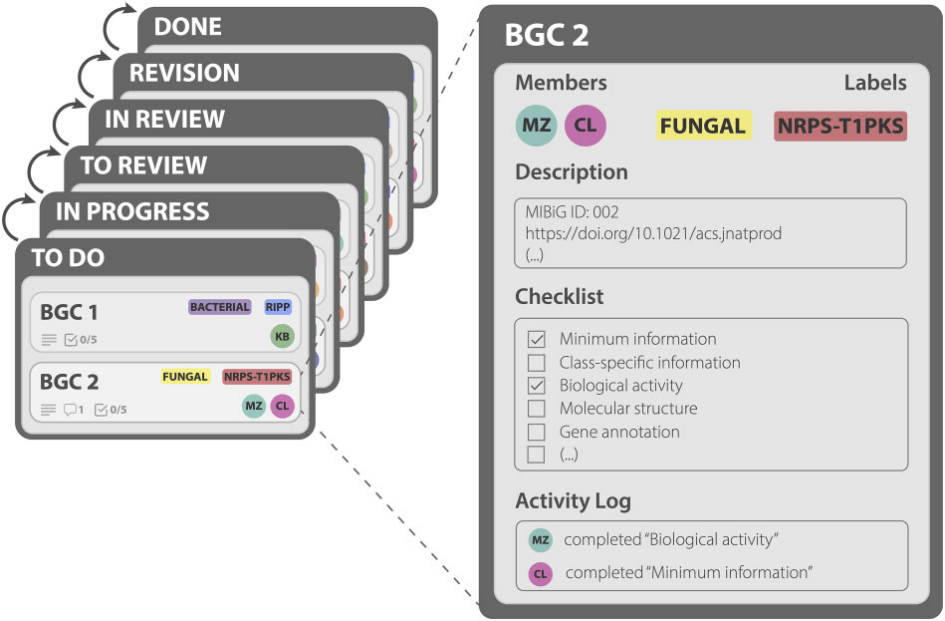
Architecture of the Kanban board used for the MIBiG annotathons. Every BGC would have its own ‘card’, where annotators with specific expertise could fill in and then check a specific part of its annotation. Once the checklist was complete, the card would move to review and, potentially, revision to repair any issues identified by the reviewers.

Inspired by the contributions made to MIBiG 3.0, we again sought participation from the scientific community. Following calls on social media, 398 researchers signed up to participate in a series of eight 3-h online annotation sessions, accommodating different time zones (Figure 1). This enormous interest posed organizational challenges in terms of coordination and communication, prompting us to develop a new model for community participation. Individual contributors were part of one or more Interest Groups that communicated using the MIBiG Slack (https://mibigannotathons.slack.com/) channel and were headed by Interest Group Coordinators: topic matter experts responsible for answering biosynthesis- and chemistry-related questions. Kanban-style boards (free version of Trello, https://trello.com/) were employed to coordinate work on entries. Data submission was performed using a MIBiG Submission Portal prototype, a bespoke web interface that uses validated fields for data processing (code available at https://github.com/nlouwen/submission-prototype). Several curators with relevant expertise volunteered to take Reviewer roles, focusing on assessing the quality of newly generated or modified entries using the newly introduced peer review system. Aimed towards further improving the quality and confidence of entries, Reviewers could leverage the Kanban-style boards (Figure 2) to request revisions of entries if errors were found. To facilitate data curation, we prepared extensive online documentation (Supplementary Data 1) and instructional videos, and trained Interest Group Coordinators and Reviewers for their roles in online meetings. Participants who made a significant contribution (defined as participating in at least two 3-h sessions or an equivalent time investment) were invited to be co-authors in the present publication.

**Figure 3.**
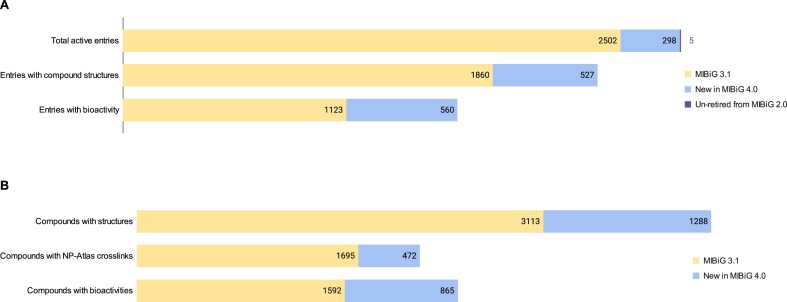
Quantitative overview of updates to the MIBiG database.in comparison with the previous version 3.1. Numbers in panel (**a**) refer to MIBiG entries, while numbers in panel (**b**) refer to individual compounds (a single MIBiG entry may contain more than one compound).

## Results and discussion

### Advancing the MIBiG data repository

In this iteration of the MIBiG annotathons, we put a greater emphasis on self-organization and facilitating motivated contributors to act independently and confidently when curating data. During the call for participation, researchers not only signed up to participate, but also contributed to assembling a list of recent publications associated with the biosynthesis of SMs. This initial effort yielded 552 publications supporting new entries and 266 publications for improvements of existing entries, which were used as a starting point for the curation process. Over the course of the annotathons, 267 contributors made a total of 8304 edits (e.g. adding an entirely new entry, adding biological activity to an existing entry, etc.), resulting in 557 new and 590 modified existing entries. With the present update, MIBiG now contains a total of 3059 entries, a 22% increase in comparison to MIBiG 3.0. Of these, 1655 entries are now associated with 3604 biological activities, and 2634 entries have 5002 associated chemical structures. However, 672 entries still lack chemical structures; hence, future efforts will include attention to improving this aspect, especially with regard to structural information for ribosomally synthesized and post-translationally modified peptides. Additionally, 7677 references and 8582 evidence qualifiers were provided, 171 biosynthetic paths were described for 110 entries and cross-references to 173 MITE and 93 CyanoMetDB entries were established. A summary of the changes in comparison to MIBiG version 3.1 can be seen in Figure 3.

Of the total 1147 contributed entries (557 new, 590 modified), 464 (40%) have been reviewed at the time of manuscript preparation. While all entries are available, those that are reviewed are highlighted in the MIBiG repository website to reflect the additional confidence. For applications using the MIBiG data where a high confidence level is required (e.g. machine learning applications), we recommend the use of reviewed entries only (the website facilitates filtering/sorting on this). We expect the ‘reviewed’ part of the MIBiG repository to grow continuously once we have transitioned to the MIBiG rolling release model, and over time, we aim to formally review all entries in the MIBiG repository.

### Initiating the MIBiG rolling release model

The aforementioned efforts demonstrate the value of leveraging large community initiatives such as the MIBiG annotathons. We estimate that contributors volunteered ∼4000 h in curating and reviewing entries, an effort in time and expertise that could not be raised by any single research group. Besides expanding the MIBiG repository, the annotathons were appreciated for their community-building aspect, fostering communication and exchange of ideas in the SM research community. In addition, the interaction with other resources prompted improvements to these databases as well, e.g. when curators could not find matching entries for a structure in the NP Atlas, thus encouraging wider cooperation beyond MIBiG itself. The broad interest of the community motivated the planning of a ‘rolling release’ model of MIBiG. In addition to the biennial efforts that will lead to ‘major’ releases of MIBiG (e.g. the current v4.0, or the next major release v5.0), curators will be able to contribute new or modify existing entries on an *ad hoc* basis, leading to quarterly ‘minor’ releases (i.e. 4.1 and 4.2). Contributors will be able to correct bugs and add references at any time, instead of waiting for the ‘major’ release cycle to perform all edits at once. This new system is currently under development, and we invite the scientific community to participate in the discussion on how to structure contributions and governance (i.e. by communicating with the corresponding authors of this publication or using the MIBiG Slack Workspace https://mibigannotathons.slack.com). Furthermore, to facilitate future MIBiG updates and curation we encourage authors to release BGC sequence data during the publication submission and peer review process, or immediately thereafter, and to provide the respective accession details in the manuscript text.

In summary, we have conducted a large-scale community effort to make experimental data on SM BGCs freely accessible and machine-readable. As a resource created for and by the scientific community, the MIBiG repository is freely accessed on an entry-by-entry basis or can be downloaded and parsed in bulk. MIBiG 4.0 also serves as the stepping stone for creating the infrastructure to establish a Wikipedia-like model of continuous community curation. Such a decentralized organization will guarantee continuous development of MIBiG and help in including the next generations of scientists in the annotation and development process.

## Supplementary Material

gkae1115_Supplemental_File

## Data Availability

The MIBiG repository is available at https://mibig.secondarymetabolites.org/. Files in JSON format following the MIBiG data standard (https://github.com/mibig-secmet/mibig-json) can be found on the MIBiG webpage (https://mibig.secondarymetabolites.org/download) and on the MIBiG Zenodo Community page (https://doi.org/10.5281/zenodo.13367755). Further materials are available on GitHub (https://github.com/mibig-secmet). All data are freely available with no restrictions for academic and commercial reuse under the OSI-approved CC BY 4.0 Open Source license (https://creativecommons.org/licenses/by/4.0/).
