# Supplementary material for: MIBiG 4.0: advancing biosynthetic gene cluster curation through global collaboration
Source: Nucleic Acids Res. 2024 Dec 9;53(D1):D678–90. doi: 10.1093/nar/gkae1115 (PMC11701617; doi:10.1093/nar/gkae1115)
Supplement: gkae1115_Supplemental_File [file gkae1115_supplemental_file.docx]

# MIBiG 4.0: Advancing Biosynthetic Gene Cluster Curation through Global Collaboration - Supplemental Information

Mitja M. Zdouc*, Kai Blin*^✝^, Nico L.L. Louwen, Jorge C. Navarro-Muñoz, Catarina Loureiro, The MIBiG Annotation Consortium, Justin J.J. van der Hooft, Roger G. Linington, Tilmann Weber^✝^, Marnix H. Medema^✝^

*** Co-first authors ✝ Co-corresponding authors

MHM: [marnix.medema@wur.nl](mailto:marnix.medema@wur.nl) TW: [tiwe@biosustain.dtu.dk](mailto:tiwe@biosustain.dtu.dk) KB: [kblin@biosustain.dtu.dk](mailto:kblin@biosustain.dtu.dk)

##

## Supplementary Data 1 - MIBiG Data curation ‘Annotathon’ protocol

[**Overview 4**](#_5tlqb9cw14ah)

[Section 1: MIBiG Overview 4](#_d00jmlkbsf8j)

[1.1 The MIBiG Database 4](#_clebmskbt4di)

[1.2 The MIBiG Entries 4](#_vldxppft8mhq)

[1.3 The MIBiG Data Standard 5](#_hae7v8t25z6x)

[**Section 2: MIBiG Entry Creation/Improvement 6**](#_py8pd5bs95lw)

[2.1 Organizational Structure 6](#_dys6x5p8f8ua)

[2.1.1 Contributors 6](#_o95bwpvf00f5)

[2.1.2 Interest Groups 7](#_scp8yckjwrpf)

[2.1.3 Interest Group Coordinators 7](#_6cgdsaa73lq4)

[2.1.4 Reviewers 7](#_p9fxsrs1simv)

[2.1.5 Code of Conduct 8](#_rzxwiq77xrun)

[2.2 MIBiG Annotathon Workflow 8](#_jon03wfxg02h)

[2.2.1 Trello Board 9](#_zh8l9fvqf2j3)

[2.2.1.1 The Boards 9](#_9oh756jgfzwj)

[2.2.1.2 The Lists 10](#_6xik8d2i3c5h)

[2.2.1.3 The Cards 11](#_bdofnx69r57j)

[Creating a card: 11](#_mx5nzj561cq3)

[2.2.2 Example Workflow 12](#_j9u3svoliinb)

[2.3 MIBIG Submission Portal 15](#_kfcdp7ad3cg9)

[2.3.1 Registration 15](#_2tfk80hdlqy5)

[2.3.2 Login Process 15](#_puj9a0j68en0)

[2.3.3 Creating new entries 16](#_bgsv5hyjlsit)

[2.3.4 Modifying existing entries 16](#_z6o1myn1p4iz)

[2.3.5 Reviewing entries 16](#_7yci9joo3n0c)

[**Section 3: MIBiG Data Collection 16**](#_ai4bzyflcmup)

[3.1 How to create a minimal MIBiG entry 16](#_nqe1uot1ioy1)

[3.1.1 Locus, coordinates and organism identifier 16](#_uynfrgk1bexh)

[3.1.2 Products/Compounds 19](#_iudhzzns6us5)

[3.1.3 Biosynthetic class 19](#_mmgt0m5x6xf2)

[3.1.4 BGC evidence 19](#_o09muzpjl13y)

[3.2 How to add biosynthetic class-specific information 20](#_pd91zwot4uhf)

[3.2.1 PKS BGCs 20](#_6ids63pi25tp)

[3.2.2 NRPS BGCs 21](#_dqalbpp0cf3x)

[3.2.3 RiPP BGCs 22](#_jn3otgd7bujz)

[3.2.4 Terpene BGCs 25](#_mi41p7zc1iux)

[3.2.5 Exotic/Other BGCs 26](#_ii9uctfljh6m)

[3.2.5.1 Saccharide 26](#_twxis53c6svm)

[3.2.5.1 Other 26](#_gesr1ulrbott)

[3.3 How to add biological activity 26](#_pe7ia7fvdvgr)

[3.4 How to add the molecular structure(s) 28](#_bhnw35lbcq04)

[3.5 How to add gene annotations: 30](#_a9g81b1umkvh)

[3.5.1 Basic gene annotations 31](#_bkgv6s4rvqz8)

[3.5.2 Advanced annotations for tailoring enzymes/maturases (MITE) 31](#_n6l5dey1e8cm)

[3.5.3 FAQ MITE 36](#_nhnm285x0bca)

[Section 4: Instructions for Reviewers 36](#_o60thg9h49tg)

[4.1 Prerequisites 37](#_9m52bxawbe3k)

[4.2 The Review Process 37](#_exnbrmv23535)

[4.3 Frequently observed Problems 38](#_qudxj1dbh1xu)

[4.4 The Revision Process 38](#_bqozomvnl8ox)

[**Section 5: Frequently Asked Questions 39**](#_kyjf5f1vw06y)

[What to do if a paper lacks even the essential information to create an entry? 39](#_xfcrltciuo0g)

[Does any specific software need to be installed before the annothatons? 39](#_4zbr7cmrw52v)

[Multiple BGCs from a single paper 39](#_yvd4kffufida)

[No compound name 39](#_i9hz634jquui)

[Minimal Evidence for MIBiG? 39](#_4lhwntjpj9ra)

[What if a paper lacks e.g. A-domain specificities? 40](#_gvpnozk6uefm)

[Automated way of submitting large in-house datasets? 40](#_610h3ht9c2pk)

[How long does it take to create one MIBiG entry? 40](#_ru5drlvwf8hf)

[Work outside of annotathon times? 40](#_3szu3x9vggn1)

[Can Reviewers also create entries? 40](#_6ajz9oy20xfx)

[What is the deadline for creating/reviewing entries? 40](#_b6cmgfpcn4vk)

[How to treat papers that are currently under submission/in revision? 40](#_ghpj4rv5vuuy)

[On the Trello board, is each card a BGC OR a publication? 40](#_tlccf4ec3q3s)

[How to deal with partially clustered/non-clustered metabolite pathways? 41](#_2e2rxsgk2lw2)

[What if a BGC misses some genes because it lies on a contig edge? 41](#_6kicn3xy9nq2)

[What if a BGC product is glycosylated, but the BGC lacks a glycosyltransferase? 41](#_605wdyv6uahy)

[For NRPS A-domain specificity, which amino acid should be added? 41](#_v7c40v2ljk17)

[Why not use large language models to extract data from publications? 41](#_3faweazd7iaw)

[For MITE, are two homologous genes/enzymes two different entries? 41](#_p45uk7y8yun)

[Is it worth adding an already present BGC from a different organism? 41](#_eyi11hnjgcty)

[**Bibliography 42**](#_q0n1vymfwncx)

## Overview

The scope of this document is to provide methods and protocols for creating new and curating existing entries for the Minimum Information about a Biosynthetic Gene Cluster (**MIBiG**) database. The document is separated into three parts.

- The **first part** gives a brief **overview** about MIBiG and its main concepts, the structure of the database and its entries.
- The **second part** gives an introduction to the **curation workflow** and a description of the different roles. It explains the use of the **Trello board** for coordination, the data input via the **MIBIG online submission system**, and the **review procedure**.
- The **third part** provides **details on data collection** for the individual elements of the MIBiG entry, and a collection of **frequently asked questions**.

## Section 1: MIBiG Overview

### 1.1 The MIBiG Database

In 2015, the MIBiG database was established as a standardized and systematic format to collect experimentally confirmed data about biosynthetic gene clusters (BGC) and their associated products [(1)](https://paperpile.com/c/TLintO/78JV). The aim of this data standard was to give researchers a freely accessible knowledgebase for comparative analysis, function prediction, and de novo design of biosynthetic pathways. Nine years and two iterations of crowd-sourced annotation efforts later, MIBIG 3.1 now holds over 2500 entries of natural product BGCs and is considered one of the leading databases on this topic [(2)](https://paperpile.com/c/TLintO/T5pp). As a community-driven effort, MIBiG is freely accessible under an open source license, and exemplifies the benefit of open science in general. The MIBiG database is maintained by the Weber group at the Technical University of Denmark and is accessible at <https://mibig.secondarymetabolites.org/>.

Given the rapidly expanding capabilities of both genome mining and synthetic biology, more and more BGCs and their products are being characterized. This provides again a great opportunity to further update the MIBiG database to version 4.0 with additional entries in a series of new community-driven annotation hackathons (also known as “annotathons”). However, contribution of data in the correct format can sometimes be challenging, due to the strict formalism of the MIBiG entries. To facilitate the interaction of contributors with the MIBiG data standard, this collection of protocols was created.

### 1.2 The MIBiG Entries

Each MIBiG entry describes a **single BGC**, characterized from a **single organism**, and its **experimentally linked products**. It is associated with a unique MIBiG identifier (starting with the letters BGC, followed by a seven digit code, e.g. BGC0000001). Each MIBiG entry exists as a single, self-contained JSON file, and follows the MIBiG JSON Schema, a type of controlled vocabulary that specifies allowed parameters and their type (see below). A MIBiG entry consists of the following groups of data collection (discussed in more detail in Part III: MIBiG Data Collection):

- **Changelog/History** (essential): each MIBiG entry tracks its creation and modification history: **who** has done **what** **when**. The changelog is updated automatically and not part of the annotation procedure, but is included here for completeness.
- **The minimal entry information** (essential): the minimal data required for the creation of a new MIBiG entry. This includes the genome identifier, the location coordinates of the BGC on the genome, associated products, and experimental evidence.
- **Class-specific biosynthetic information** (optional): for each biosynthetic class, additional class-specific information can be provided. Currently accepted biosynthetic classes are *non-ribosomal peptide synthetase* (NRPS), *polyketide synthase* (PKS), *ribosomally synthesized and post-translationally modified peptides* (RiPPs), *terpenes*, *saccharides* and *other* (an umbrella class for less prevalent biosynthetic classes). For simplicity, during the annotathons, *saccharides* and *other* are grouped into the group *Exotic*.
- **Biological activity information** (optional): summarizes biological activity information about the products associated with the BGC, for example antibiotic activity.
- **Chemical structures** (optional): collects chemical structures and other compound-specific information, including cross-links to databases such as NPAtlas.
- **Gene Annotations**  (optional): Gene specific annotations such as functional information and cross references to related databases such as the Minimal Information about a Tailoring Enzyme (MITE) database. Newly introduced in MIBiG 4.0, MITE is an associated but distinct data standard specifying substrate specificities and reactions of tailoring enzymes (maturases) associated with the BGC. These tailoring enzymes often introduce modifications to the core scaffold that are essential for the observed biological activity. [REF TBA]

Ideally, a MIBiG entry should summarize all available experimental data about a BGC and its associated products, as well as cross-reference to other, specialized databases, such as UniProt for proteins or NPAtlas for details on chemical structures. Unfortunately, many MIBiG entries are incomplete, which is a disadvantage for its purpose as knowledge base, but also for other applications that link to it (e.g. antiSMASH) or use its data for machine learning purposes. Therefore, **improving existing entries** is at least as important as adding new entries.

### 1.3 The MIBiG Data Standard

All MIBiG entries are based on the MIBiG data standard, which was first specified in the original MIBiG publication [(1)](https://paperpile.com/c/TLintO/78JV). It has become customary that every MIBiG iteration is accompanied by adjustments to the MIBiG Data Standard. The most recent changes accompanying MIBiG 4.0 include:

- More accurate tracking of evidence (literature references) for individual components of the entry (i.e. evidence reference for e.g. the biological activity of a compound must be specified explicitly.
- Addition of pathway descriptions
- A rework of tailoring enzymes, their reactions, and substrate specificities (covered by the newly established MITE data standard).
- Introduction of quality levels for entries (questionable, low, medium, high)
- Separation of biosynthetic and chemical information

With each new iteration of MIBiG, all new entries follow the most up-to-date data standard, and old/existing entries are updated.

## Section 2: MIBiG Entry Creation/Improvement

Given the great variety of experimental data it accommodates, MIBiG needs to employ a high degree of formalism. Furthermore, as a crowd-sourced project, the MIBiG Annotathons unite a large number of researchers with different expertises and working styles. This makes the direct interaction with the MIBiG data standard challenging. To address this problem, the MIBiG team has invested into the preparation of organizational frameworks, methods, and protocols to facilitate and unify the creation/modification of MIBiG entries.

### 2.1 Organizational Structure

The MIBiG 4.0 Annotathons see the introduction of a new organizational structure to facilitate interactions between participants. The most important changes are the re-definition of MIBiG **Contributors**, and the introduction of **Interest Groups,** **Interest Group Coordinators**, and **Reviewers**.

#### 2.1.1 Contributors

All active participants of the MIBiG Annotathons are considered **Contributors** (previously known as Annotators, Curators, or Participants). Contributors contribute with their time to the MIBiG database, be it by creating new entries, amending or correcting existing entries, reviewing, or coordinating other Contributors.

**All Contributors who participate in at least two three-hour annotathon sessions (or make a likewise contribution corresponding to six hours of work) will become co-authors of the MIBiG 4.0 publication.**

MIBiG is licensed under a Creative Commons Attribution 4.0 International License (https://creativecommons.org/licenses/by/4.0/). By participating in the MIBiG 4.0 Annotathons, **Contributors agree to license their contributions under the same terms**. Each single MIBiG entry tracks its creation and consecutive modifications with an appended changelog, including the identity of the Contributors by name and MIBiG user ID. During sign-up, Contributors may opt out of having their name resolved in the MIBiG entries and on the MIBiG website. However, their (anonymous) MIBiG user ID will still be tracked.

#### 2.1.2 Interest Groups

Interest Groups organize individual Contributors with similar interests (e.g., NRPS substrate specificity). Interest Groups are non-exclusive, meaning that a single Contributor can be part of many Interest Groups. However, every Contributor must be part of at least one Interest Group. The Interest Groups provide scientific advice, facilitate communication ( “Who is doing What?”) and help prevent redundant work. All Interest Groups have their own Slack Channel in the MIBiG Annotathons Slack Workspace (to register, follow this link: <http://tinyurl.com/mibig-slack-registration>). During the Annotathon sessions, Interest Groups have their own Zoom Breakout Room, for informal communications. Interest Groups are also intended to motivate self-organization: for example, if many people are interested in a specific topic (e.g. trans-AT PKS), a new Interest Group can be easily established, and only requires appointment of an Interest Group Coordinator (see below) and a notification of the MIBiG organizational team. However, to prevent fragmentation, an Interest Group should only be created if there are **five or more** people predominantly working on this topic.

#### 2.1.3 Interest Group Coordinators

Each Interest Group is coordinated by one or more Coordinators, who have received specific training in MIBiG annotation and error-handling and are often also specialized in the topic of each Interest Group.

Interest Group Coordinators are responsible for chairing the Interest Groups during the session, to facilitate communication and coordination, and to serve as first contact-point for topic-related or technical questions. Coordinators are also well-acquainted with the content of the present document and can refer Contributors to individual instructions.

Interest Group Coordinators have their own Slack channel to be able to coordinate between themselves. Coordinators are also responsible to moderate interactions inside the Interest Groups and report behavior that goes against the Code of Conduct (see below). For the fulfillment of their role, Interest Group Coordinators are a more prominent role in the MIBiG 4.0 publication author list, are displayed on the MIBiG website, and receive an honorary mention in the Acknowledgements. A list of all Interest Group Coordinators can be found here: [MIBiG 4.0 Interest Group Coordinators](https://docs.google.com/spreadsheets/d/1wLbf0TYxWzWxWokFYzBAx6CF_q852jUoiHmu6tJemZ0/edit?usp=sharing)

#### 2.1.4 Reviewers

The MIBiG 4.0 Annotathons see the introduction of the Reviewer as a new organizational role. Reviewers are Contributors who have volunteered to review entries created or modified by other Contributors. Usually, they are experts in the given topic and scrutinize newly created or modified entries for plausibility and scientific soundness. Reviewers may request revisions to entries that contain erroneous or incorrect data, and provide constructive feedback for their improvement. A list of registered Reviewers, including their expertises, can be found here: [MIBiG 4.0 Annotathon Reviewers](https://docs.google.com/spreadsheets/d/1SivNzlKShLTmIX-9wcWR7qz_iU6Vp83fjmKqsw5L6fw/edit?usp=sharing)

#### 2.1.5 Code of Conduct

Like every community-driven effort, the MIBiG Annotathons follow a set of rules for harmonious and productive collaborative work. During the generation and modification of entries, data is scrutinized and critically reviewed. Here, opposing views and opinions may lead to arguments and discussions, which is a normal part of the scientific discourse. Such debates are expected to be conducted gracefully, objectively, and constructively, following the guidelines established by the [Contributor Covenant](https://www.contributor-covenant.org/version/2/1/code_of_conduct/) **Code of Conduct.** These guidelines aim to make the MIBiG Annotathons a pleasant and harassment-free experience for everyone involved. Adherence to these guidelines will be monitored by the Interest Group Coordinators. In the unlikely event that undesired behavior was detected, the MIBiG organizational team will take appropriate measures.

### 2.2 MIBiG Annotathon Workflow

All MIBiG entries are based on primary literature, which provides the experimental evidence for connecting a biosynthetic gene cluster with its product(s). During the MIBiG Annotathons, new entries are created or existing ones modified, in a process which is entirely based upon published literature containing experimental data.

As such, to allow for efficient creation/modification of entries during the Annotathons, a pre-generated literature collection is required. This collection has been generated in a collaborative effort (<http://tinyurl.com/mibig-literature>), and automatically processed to generate the Trello cards (see [below](#kix.odbpyfe0tuov)) that guide the annotation effort

For an introduction on how to use the Trello boards together with the MIBiG Submission Portal, see this video: <https://youtu.be/dj2DVcL7aTM>

During the Annotathons, Contributors will generate/modify entries. These entries will be reviewed by Reviewers, in an iterative process that ultimately leads to the approval and submission of a given entry to the database (Fig.1). Both generating/modifying entries and reviewing these entries is done using the [MIBiG Submission Portal](#kix.68vy657v1zhg) web interface.


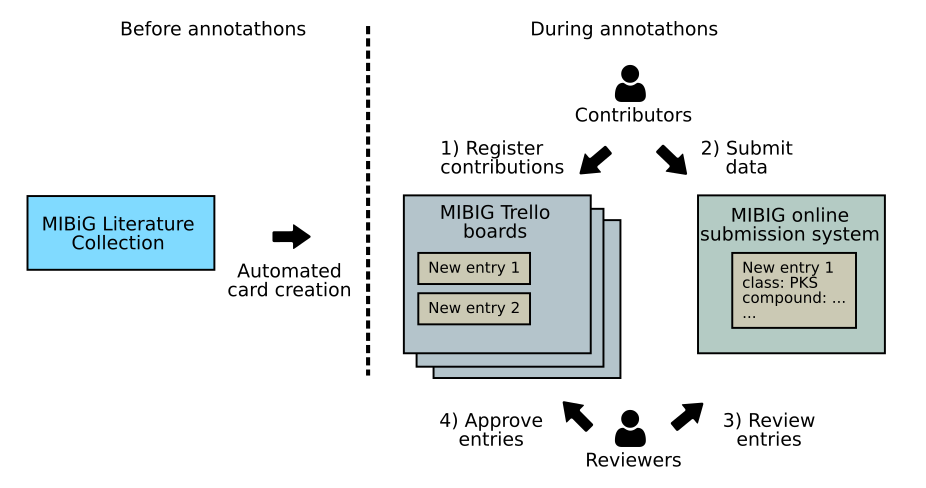


Figure 1: MIBiG Annotathons workflow schema

#### 2.2.1 Trello Board

In collaborative work, it is often challenging to coordinate **who** is currently doing **what**. In MIBiG, this issue is addressed by using a [Trello](https://trello.com/) board. A Trello board provides an interface where tasks and their progress can be visualized graphically (Fig.2). The MIBiG Trello boards follow the [Kanban principle](https://en.wikipedia.org/wiki/Kanban): they are divided into several columns (=Lists) representing steps in the curation workflow. These Lists contain cards which represent a single new or existing MIBiG entry. During the curation work, the cards are moved through the board from left to right, until they are completed and the full entry is created.

All MIBiG Coordination Trello boards are public, meaning that everybody on the internet can see their content. However, to add or modify entries, one must be a member of the Trello board. All people who registered for the MIBiG 4.0 Annotathons will be added to the boards automatically; if not, contact one of the board administrators. Access to the premium version of Trello is **NOT** required.

**Update March 2024**: Trello is taking steps towards limiting the number of users for a Trello board (before: unlimited). However, these changes will become effective only **AFTER** the MIBiG 4.0 Annotathons have been conducted. For the MIBiG 4.0 Annotathons, we will proceed as planned.

For an introduction on how to use the Trello boards together with the MIBiG Submission Portal, see this video: <https://youtu.be/dj2DVcL7aTM>

##### 2.2.1.1 The Boards

Based on the organism of origin of the BGC, different MIBiG Trello boards exist for organizational reasons:

- Bacteria (BGCs of bacterial or archaeal origin):
  <http://tinyurl.com/mibig-coordination-bacteria>
  (responsible person: Mitja M. Zdouc)
- Micro-Eukaryotes (BGCs of fungi, yeasts, protists, microalgae):
  <http://tinyurl.com/mibig-coordination-micro>
  (responsible person: Jorge C. Navarro-Muñoz)
- Macro-Eukaryotes (BGCs of plants, animals, macroalgae):
  <http://tinyurl.com/mibig-coordination-macro>
  (responsible person: Elena del Pup)


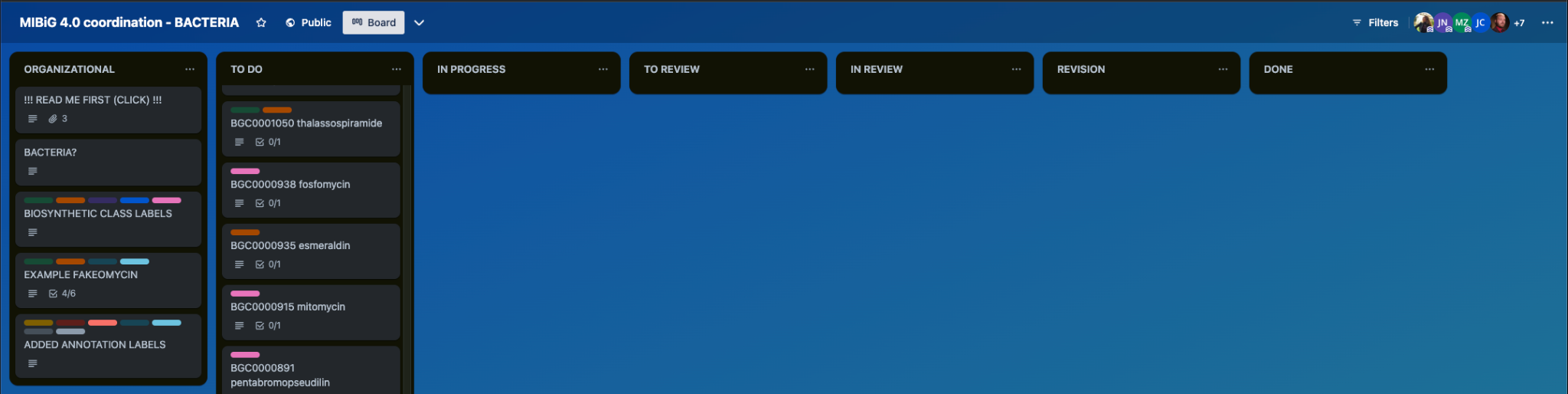


Figure 2: Overview of the MIBiG Trello board for coordinating BGCs from bacterial^^[[1]](#footnote-0)^^ origin.

##### 2.2.1.2 The Lists

Every Trello board has a number of columns (known as Lists) to organize the information flow (Fig.2):

- **Organizational**: This List contains a cross-reference to the curation protocol (README), an example BGC card (*Fakeomycin*), as well as cards describing the Biosynthetic Class and Annotation labels.
- **To Do**: Each card represents a new or existing MIBiG entry and tracks its annotation progress. **Cards are always moved from left → right.**
- **In Progress**: When annotation work for an entry starts, the card is moved to the **In Progress** List and assigned to a Curator (which can be yourself). Curators can either do parts of or the complete annotation work. After each annotation step, labels indicating the type of added data are added to the card. This allows to quickly see the completeness of the entry. Labels should never be removed (e.g. biosynthetic type).
- **To Review**: Once all annotation work is finished for an entry, its card is moved in the **To Review** List. From here, a Reviewer can pick up a card and move it to the **In Review** list.
- **In Review:** In this List, cards are placed that are undergoing review. Multiple Reviewers can work sequentially on a card. Once Review is finished, the card is moved either to Revision, or to Done, depending on the outcome of the Review.
- **Revision:** If the review process detects any major errors, the card is moved to the List **Revision** in which it resides until errors are fixed.
- **Done:** If no errors were detected during review or all errors have been fixed, the card is moved to the List **Done**.

##### 2.2.1.3 The Cards

There are two card types:

- New Entry
  - Name/id: compound/BGC name
  - Description: MIBiG UID, producer organism strain, DOI(s) of relevant literature, any notes.
  - Checklists for Contributors and Reviewers
- Existing Entry
  - Name/id: MIBiG BGC identifier (BGC000****) + compound name
  - Description: MIBiG UID, overview of issues to be fixed.
  - Checklist: concrete tasks required to fix each issue.

Both card types are available as **Templates** (see below). **New Entry** cards represent MIBiG entries that are newly created. These cards need more preliminary information and effort to add the minimum required data. **Existing Entry** cards represent additions or modifications to existing entries and assume the minimum required data for an entry is already present. Therefore, these cards are easier to create and complete.

##### Creating a card:

At the bottom of each List, on the right side, a **small icon** can be found. Click on it to access the Templates (Fig.3). Then, the correct card template can be accessed with a click. Once the card is created, click on it to update the name, description and fill the required information.


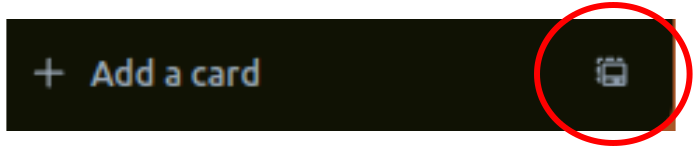


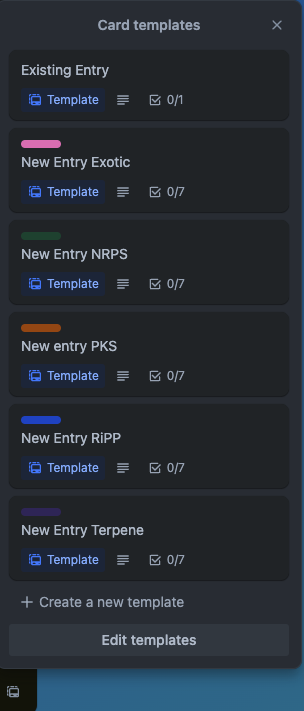


Figure 3: the card Template menu.

| **Infobox** *Expanded card labels and colorblind friendly mode*  On the Trello board, card labels show information about the status of the entry at one glance. However, in default mode, these labels are difficult to recognize, and are not colorblind friendly. However, this can be fixed in a few steps  How to expand the labels and add colorblind friendly mode  In any card (Figure 4A), click on one of the labels. This will expand the labels, adding the title text (Figure 4B). Open the card, go to “Labels”, scroll to the bottom of the pop-up menu, and click on **“Enable colorblind friendly mode”.** This will add additional patterns to the cards, which can make it easier to distinguish the individual labels.  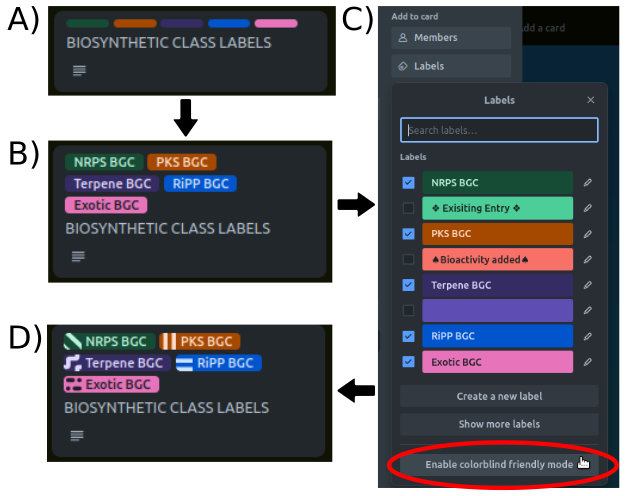  Figure 4: Instructions to enable large labels and colorblind friendly mode in Trello. |
| --- |

#### 2.2.2 Example Workflow

In this hypothetical scenario, we assume that we are working on a new entry for the hybrid NRPS/PKS BGC [fakeomycin](https://trello.com/c/Y8fZjwq9/11-example-fakeomycin) from *Streptomyces nonexistensis*.

1. Before starting, we check to see whether a card for fakeomycin already exists on the board. The board can be searched with the Filters button on the top right side. Also, the MIBiG repository must be searched for fakeomycin from *Streptomyces nonexistensis*. Let us assume that we only find fakeomycin from ***Nocardia*** *nonexistensis -* this is a different Genus, so we can still create a new entry for fakeomycin for *Streptomyces nonexistensis*.


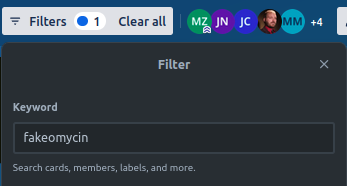


1. Since no card is present, and no MIBiG entry is available, we create a New Entry card. In the NRPS and the PKS Interest Groups, we briefly announce which entry we work on (it is possible that two contributors simultaneously intend to create a card for the same BGC). The card for the fakeomycin BGC is first created in the List TO DO using the New Entry NRPS template and filling in the mandatory data. Since it is a hybrid BGC, the label PKS BGC needs to be added manually (if the card had been created using the New entry PKS template, the label NRPS BGC would have been required).


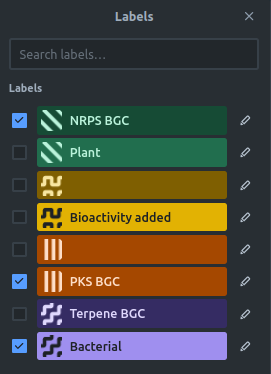


1. Using the Add Member function of the card, we assign ourselves to it, and move the card to the **IN PROGRESS** List. Then, we create the entry using the [MIBIG Submission Portal](#kix.68vy657v1zhg) web interface. **Once this entry is created, we get a temporary MIBiG Unique Identifier (UID), and add this UID to the Trello Card Description. This allows us and other people to find the card again**. We can also add specific comments about any special steps taken to gather the data.
2. Let’s assume we first only add the minimum information needed for an entry. When finished, we only tick off the **minimum entry data checkbox** of the card, add the **Minimal added** label, and unassign ourselves from the card again. Depending on the card, the checklist may also be in the description of the card instead.

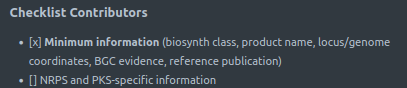


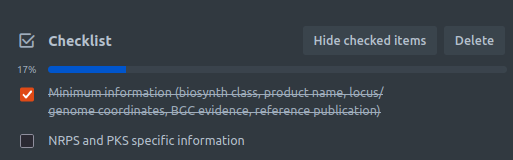

3. Later, we find another paper that describes the NRPS and PKS specific information about the fakeomycin BGC from *Streptomyces nonexistensis*. We filter for the fakeomycin card again, see that it is still unclaimed, and claim the card again by adding ourselves to it (if the card was claimed by someone else, we would need to coordinate with this person, for example using Slack - no two Contributors can work on one card at the same time). Then, we use the stored **MIBiG Identifier (UID)** to open the corresponding entry in the **MIBiG Submission Portal**. Then, we add the NRPS and PKS-specific information. We go back to the card, tick off the **NRPS and PKS specific information** checkbox, add the **Type-spec. added** label, add a comment to the activity log if necessary, and unassign ourselves again.

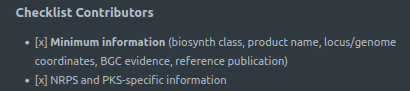


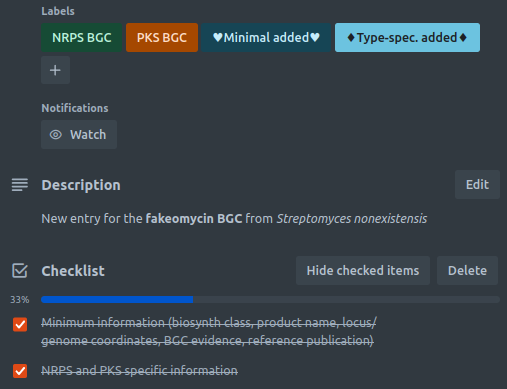

4. After a while, we see that all information is added for this card and that all checkboxes are ticked off. It can also be that the information available about this BGC is limited and that not all checkbox items can be filled. In the latter case, the non-ticked checkbox items can also be removed. We then move the card to the **TO REVIEW** List.
5. A Reviewer sees the unassigned card in the **TO REVIEW** List and assigns themselves, moving the card to the **IN REVIEW** List. They perform the review and see that the NRPS and PKS-specific information is not correct. Fixing this problem would take quite some time, which justifies a revision of the entry. Therefore, the Reviewer moves the card into the **REVISION** List and contacts us about the problem. The reviewer adds us to the card. Via Slack, the Reviewer clarifies the reason for the revision, and instructs us on how to fix the issue. We follow the suggestions and modify the entry accordingly. We then inform the Reviewer about the changes.
6. Once the mistake is fixed, the Reviewer adds their approval, and moves the card to the List **DONE**, indicating the completion of the entry.

### 2.3 MIBIG Submission Portal

The MiBiG Submission Portal is one of the major changes introduced in MIBiG 4.0. It provides a high level interface for interaction with the MIBiG data standard/repository. It allows users to create new entries or modify existing entries using a series of input forms. Therefore, direct interaction with the underlying MIBiG data standard is not necessary. Furthermore, the MIBiG Submission Portal also replaces any other input systems that were used in previous annotathons (e.g. spreadsheets). Access to the MIBiG Submission Portal will be given via email to all registered participants.

For an introduction on how to use the Trello boards together with the MIBiG Submission Portal, see this video: <https://youtu.be/dj2DVcL7aTM>

#### 2.3.1 Registration

Before starting to work on entries, Contributors must register in the submission portal. During registration, the name, the [ORCID](https://orcid.org/), and a valid Email-address must be provided. A secure password must be set. Also, if a Contributor has volunteered to act as a Reviewer, reviewing access can be requested, which will be approved by the administrator. The registration assigns each Contributor with a unique MIBiG user ID, which is used to sign every contribution a Contributor makes. During registration, Contributors can opt out of having their MIBiG user ID resolved (i.e. made public), which will keep their contribution anonymous. **This opt-out is ONLY possible during registration.**

#### 2.3.2 Login Process

After registration, Contributors can log in to the MIBiG Submission Portal. Login is required before entries can be created, modified, or reviewed. The login is performed by providing the Email address and the previously set password. This will redirect to a new page, where entries can be created, modified, or reviewed.

#### 2.3.3 Creating new entries

To create a new entry, click on Submit new entry. This will redirect to a new page with several options, and also display the **temporary MIBiG Unique Identifier (UID)** of the entry. **This UID is very important and must be added to the card of the entry on the Trello board; otherwise, this entry cannot be found by other Contributors or Reviewers.** The minimum information required to create an MIBiG entry can be added by clicking on the “Minimal Entry” button. Follow the instruction in Part III of this protocol: [How to create a minimal MIBiG entry.](#kix.38u61oahfnyh) Feel free to also provide additional information (e.g. information about the structure).

#### 2.3.4 Modifying existing entries

Using the **UID** of the entry, a previously created entry can be accessed again for further modification. Remember to tick off the respective checkbox items in the corresponding Trello card.

#### 2.3.5 Reviewing entries

Using the **UID** of the entry, a previously created entry can be accessed for review. More information can be found in the [Instructions for Reviewers](#kix.lsar2fl0c1m) part of the protocol.

## Section 3: MIBiG Data Collection

MIBiG entries consolidate various data from primary literature (for an overview of the individual parts of an MIBiG entry, see [The MIBiG Entries](#kix.on1diulf0sy5))**.** This data is sometimes difficult to obtain and to interpret. In this section, protocols and instructions are provided to facilitate this procedure and to unify approaches.

### 3.1 How to create a minimal MIBiG entry

This section discusses the minimum information necessary to create a MIBiG entry.

#### 3.1.1 Locus, coordinates and organism identifier

The core information of a MIBiG entry is the **genomic locus** - the stretch of DNA where the BGC is located. In some cases, multiple loci can constitute the biosynthetic path of a NP (e.g. many plant secondary metabolites). MIBiG accepts such partially clustered BGC **as long as the majority of the BGC is physically clustered in one location, and only satellite genes from other parts of the genomes are recruited.** Unclustered pathways are **outside** the scope of MIBiG (also see the FAQs).

**Each locus consists of two components: the genome identifier and the start and stop coordinates that define the respective stretch of DNA. Without this information, MIBiG entries cannot be created!**

Here is the protocol on how to add this information:

1. From the publication, obtain the genome identifier (the accession id). This must be a GenBank accession (see the infobox **“GenBank vs other NCBI identifiers”** below)
   1. Check if there is a NCBI Nucleotide entry link, a GenBank accession, or a RefSeq accession number. This information can often be found in the Methods or Data Availability (Supplementary Information) section.
   2. If no NCBI Nucleotide entry is found in the paper, check if there is a specific genome sequence named in the paper, or locus tags that are unique to a specific genome.
   3. If no specific genome sequence was indicated in the paper, check if there is a genome sequence available with the name of the organism mentioned in the paper. Make sure that the **strain identifier** matches as well, not just the species (e.g. *E. coli* **Nissle 1917** and *E. coli* **CFT073** are **not equivalent**). Try finding the BGC in the genome sequence (e.g. in antiSMASH-DB, or running a new antiSMASH job).
   4. If all these steps do not lead to an unambiguously identifiable BGC, then **this paper cannot be used as reference** for the BGC without contacting the authors and asking about which genome sequence was used. This may not always be feasible. In such cases, add the label **“CRITICAL ERROR”** (also see FAQs) and contact one of the Interest Group Coordinators. They will decide on how to proceed further.
2. Obtain the locus coordinates (the BGC “start” and “end” sites).
   1. If the GenBank nucleotide entry contains only the BGC, the complete entry can be referenced (bases 1 to n, where n is the length of the record). Any flanking regions can be included too. For an example, see <https://www.ncbi.nlm.nih.gov/nuccore/MW201789.1?from=1&to=1920>.
      1. Ensure that additional unrelated genes are not included in the entry. For example, the “[albomycin biosynthetic gene cluster](https://www.ncbi.nlm.nih.gov/nuccore/JN252488.1)” record contains seven additional ORFs that are not part of the cluster in the [publication](https://www.jbc.org/article/S0021-9258(23)00215-6/fulltext).

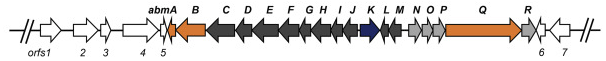

   2. If the GenBank nucleotide entry constitutes a whole chromosome or a scaffold/contig from a genome assembly, the coordinates have to be found manually:
      1. If the coordinates are mentioned in the paper, use these as start/end sites.
      2. If locus tags are mentioned, find the corresponding genes in the nucleotide entry (GenBank file) and identify the gene cluster start/end sites based on this.
      3. Sometimes the only thing to go by is a picture of an assembly line or gene arrows. In such a case, you can sometimes still find the gene cluster by comparing the picture with antiSMASH predictions from antiSMASH-DB or a custom antiSMASH run on the genome. Furthermore, the NCBI Genome Browser can be used to identify the putative BGC (Fig.5).
3. Indicate the literature reference and evidence for the entry.
   1. Literature reference: the reference of the publication. Ideally a DOI, but PubmedID or Google Patent URL are also accepted. Pre-prints are generally accepted as literature reference, as long as they provide the necessary evidence.
   2. Evidence: Indicate the type of evidence that links the BGC to its products (e.g. heterologous expression). Multiple types of evidence can be specified.


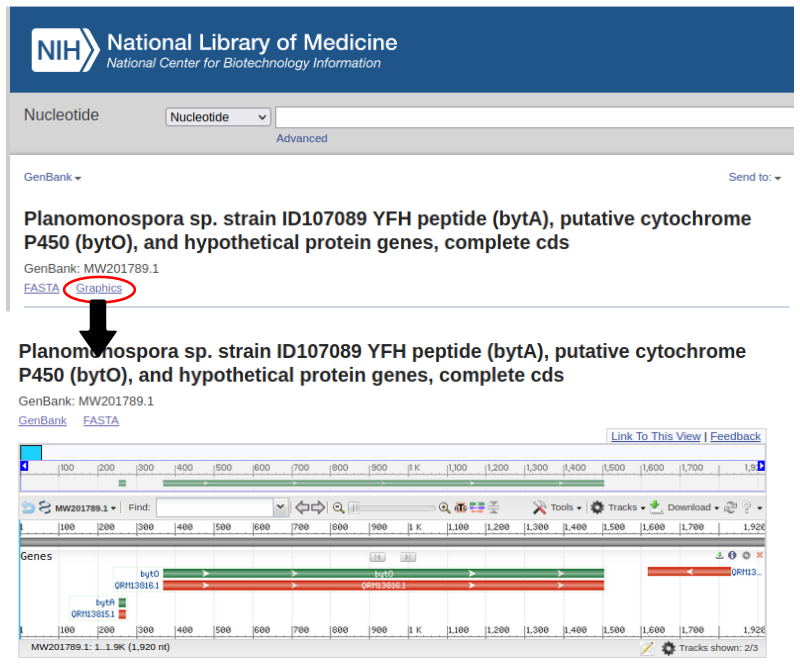


Figure 5: The NCBI Genome browser

| **Infobox** *GenBank vs other NCBI identifiers:*  NCBI hosts a variety of databases with different purposes. One of these databases is the GenBank database. For MIBiG entries, only GenBank accessions are accepted, since it is a permanent reference to the genome sequence. Other NCBI databases, such as RefSeq, frequently change genome annotations and retire sequences, making them unusable as permanent references.  How to recognize a GenBank accession identifier:  GenBank accession identifiers start with one or more capital letters, followed by several digits, a dot, and further digits. The dot and consecutive digits indicate the version of the record and may be omitted.  What are illegal identifiers?   - Containing an underscore (“_”): a RefSeq record (see *“Change RefSeq to GenBank”*) - "WP_…" or "YP_…": a RefSeq protein identifier - “GCF_…” or "GCA_…": an assembly ID - "SRR…" or “SRA…”: a SRA record   Change RefSeq to GenBank:  RefSeq entries are usually derived from GenBank entries and therefore, a RefSeq ID can be turned into a GenBank ID quite easily. Usually, it is sufficient to drop the prefix of the RefSeq ID to arrive at the GenBank ID (e.g. RefSeq ID NZ_BOOL00000000.1 corresponds to GenBank BOOL00000000.1). However, gene annotations may be different between GenBank and RefSeq, so it is necessary to double-check. |
| --- |

#### 3.1.2 Products/Compounds

In each MIBiG entry, a BGC must be linked to its product(s) by experimental evidence. The evidence linking the BGC to the product(s) is described under “Loci” (see “Locus and coordinates” above). Besides this evidence, also the product/compound names themselves must be added. Additional information, such as their chemical structure and the evidence that was used to determine them can be found under [How to add the molecular structure.](#kix.buxxkuxdo9ht)

1. Search the publication for the product/compound name(s). If the BGC has multiple (main) products, please report all of them (e.g. Erythromycin A, Erythromycin B; **not** **‘erythromycins’**) .

#### 3.1.3 Biosynthetic class

The type of each BGC needs to be classified as one or more (for hybrids) types of the following biosynthetic classes:

- NRPS (Non-ribosomal peptide synthase)
- PKS (Polyketide synthase)
- RiPP (Ribosomally synthesized and post-translationally modified peptides)
- Terpene (Terpene synthases)
- Saccharide
- Other

#### 3.1.4 BGC evidence

The evidence for the connection between the BGC and its product(s) must be one or more of the following types.  **Stronger forms of evidence, listed first, are preferred.** Please include all forms of evidence that are present in the publication(s).

- In vitro expression
  - An entire biosynthetic pathway is reconstructed using purified biosynthetic enzymes outside a living cell, and the metabolite(s) are produced. This is considered unequivocal proof.
- Heterologous expression
  - The complete biosynthetic pathway is cloned into a non-producing host, and the metabolite(s) are produced. This is likewise unequivocal; however the biosynthesis may require missing satellite genes that are complemented by the host genome.
- Enzymatic assays
  - A portion of the biosynthetic enzymes are studied experimentally. Examples include in-vitro or heterologous expression of single enzymes. This form of evidence is weaker because it does not ensure that all biosynthetic genes have been identified.
- Knock-out studies
  - Deletion or inactivation of biosynthetic gene(s) in the pathway totally stops production of the metabolite(s). While this is good evidence, it is possible that altering one pathway disrupts another through a regulatory link.
- Gene expression correlated with compound production
  - Across multiple environmental conditions, higher expression of genes in the BGC (measured by RNA-Seq, blots, qPCR etc) is correlated with higher production of the metabolite(s). A weak form of evidence, as correlation does not necessarily imply causation.
- Correlation of genomic and metabolomic data
  - A BGC is reported and metabolite(s) consistent with the BGC were characterized experimentally in the same strain. This is the weakest form of accepted evidence, as the BGC and metabolite(s) may be unrelated. Ideally, the product was characterized with analytical methods beyond mass spectrometry, such as MS/MS, UV, NMR, or Marfey’s analysis.
- Homology-based prediction (unacceptable for MIBiG 4.0)
  - A BGC is predicted, but the associated metabolite(s) were not characterized experimentally in that strain. Publications reporting **only** homology-based predictions are **not suitable** for MIBiG. This evidence grade exists only for backwards compatibility with existing entries; most entries with this evidence level will be re-labeled with “Correlation of genomic and metabolomic data”.

### 3.2 How to add biosynthetic class-specific information

#### 3.2.1 PKS BGCs

*Note: this description is still incomplete and may be expanded in the future. In case of questions, please contact the PKS Interest Group Coordinators.*

For PKS BGCs, add the following information:

1. Specify the sub-class of the PKS. Valid options are:
   1. Type I
   2. Type II aromatic
   3. Type II highly reducing
   4. Type II arylpolyene
   5. Type III
2. Specify the cyclase gene(s) as a comma-separated list of either GenPept IDs, locus tags, or gene names.
3. Specify the ketide length.

#### 3.2.2 NRPS BGCs

*Note: this description is still incomplete and may be expanded in the future. In case of questions, please contact the NRPS Interest Group Coordinators.*

For NRPS BGCs, add the following information:

1. Specify the sub-class of the NRPS. MIBiG is following the NRPS classification scheme proposed by Dell et al [(3)](https://paperpile.com/c/TLintO/ekQ3). Valid options are:
   1. Type I (Canonical modular NRPS)
      1. **Has condensation** domain(s), **has thiolation** [T/CP] domain(s) and is **modular**
   2. Type II (Canonical non-modular NRPS)
      1. **Has condensation** domain(s), **has thiolation** [T/CP] domain(s) and is **non-modular**
   3. Type III
      1. **Has thiolation** (T/PCP) domain(s) but **not condensation** domain(s) and is **non-modular**
   4. Type IV
      1. Has **no thiolation** [T/CP] domain(s), has **no tRNA** substrate
   5. Type V (tRNA-dependent CDPS and peptidyltransferases)
      1. Has a **tRNA** substrate
   6. Type VI (NPAA clusters)
      1. **Has thiolation** (T/PCP) domain(s) but **not condensation** domain(s) and **is modular**
2. Specify the release type. Valid options are: Claisen condensation, Hydrolysis, Macrolactamization, Macrolactonization, None, Other, Reductive release. One or more release types can be specified.
3. Specify the involved thioesterases differentiating between Type I and Type II thioesterases. One or more thioesterases can be specified.

Additional information:

1. If an epimerization domain is present, and the monomer in the structure is a D-amino acid, make sure to report the specificity as an L-amino acid
2. Similarly, if a methylation domain is present, and the monomer in the structure is (N-)methylated, report the unmethylated specificity.
3. When dealing with non-proteinogenic amino acid substrates, check if tailoring happens pre- or post-assembly. Knockout experiments of other (precursor) genes in the cluster often give insight into this.
4. If the evidence is based on structural inference only, verify carefully that the molecule has actually been identified from this very strain (and not from a related organism).

#### 3.2.3 RiPP BGCs

As with the other types of biosynthetic-class, RiPP BGC-specific information is optional. However, if information is added, it must include at least i) the precursor encoding gene-identifier (step 1), the RiPP subclass (step 2), the core peptide sequence (step 3), and the corresponding evidence (publication and evidence code).

1. Provide the precursor-encoding gene identifier. Since such genes are usually short, the NCBI gene detection algorithm often fails to detect them. In such cases, the gene must be added to MIBiG manually, by providing the start and stop coordinates, and the directionality (forward or reverse). In this example, we are looking at the BGC for nocapeptin, and specifically for the genes nopA and nopB from *Nocardia terpenica* IFM 0406 [(4)](https://paperpile.com/c/TLintO/yEIe). In this protocol, we will focus on finding the location of nopA. From the publication, we know the sequence of the nopA precursor peptide, its location (upstream of the lassopeptide cyclase gene nopC), and its directionality (forward) - see Figure 6.

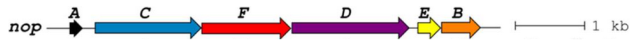

   Figure 6: the nocapeptin BGC (taken from [(4)](https://paperpile.com/c/TLintO/yEIe))
   1. First, we search the NCBI Database for the lassopeptide cyclase NopC. In the Supplementary Information of the publication, we find the RefSeq protein accession ID of NopC (WP_171983240.1), which we can search in the NCBI Protein Database.

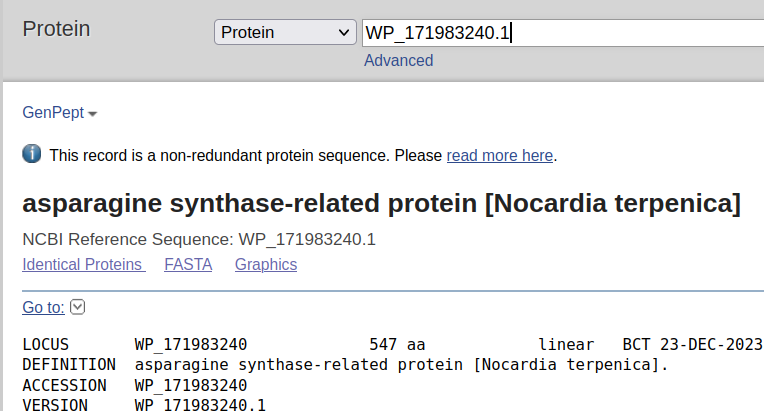

   2. Since this is a RefSeq entry (a non-redundant protein sequence) and therefore, “isolated” from its genomic context, we cannot locate NocA right away. Before, we have to find the corresponding gene in the correct GenBank sequence. To do so, we click on “Identical Proteins”.

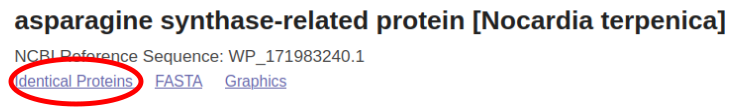

   3. On the following page, we see the references to the identical proteins. Here, we have to make sure to select the “CDS Region in Nucleotide” of the correct strain (i.e. IFM 0406).

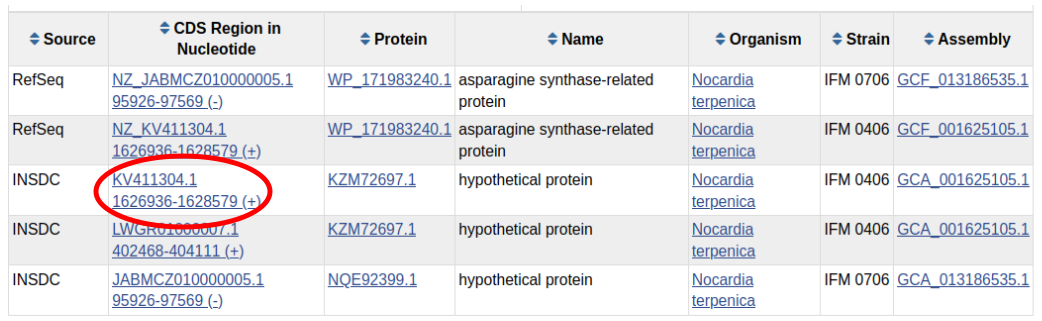

   4. On the following page, we can now change to the Genome Browser view.

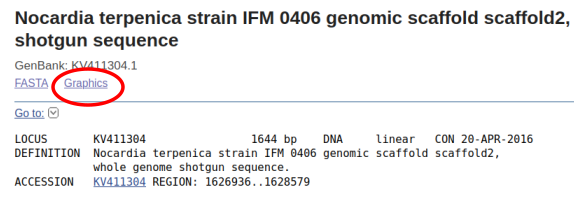

   5. This will open the Genome Browser zoomed in on the nopC sequence. Turn on the 6-frame translation by **Tracks** -> **Configure Tracks -> Sequence -> Sif-frame translation** and confirm by clicking on **Configure**.

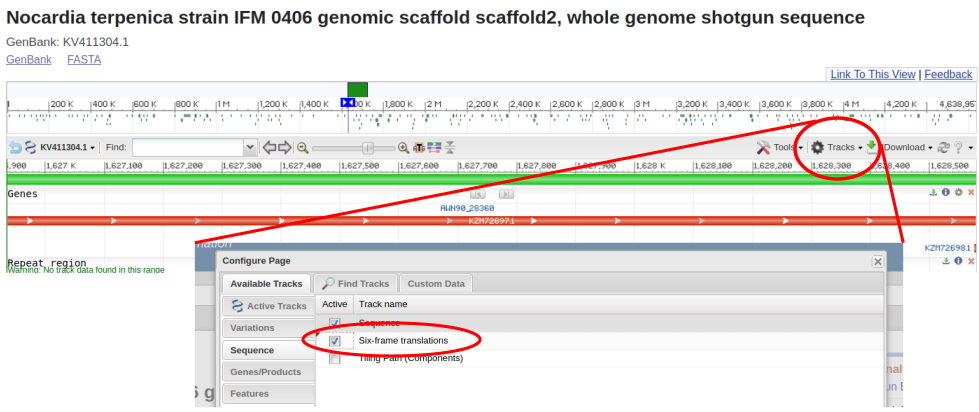

   6. Now, we have to search for the nocA CDS upstream of nocC. This is easiest by using the zoom and location arrow.

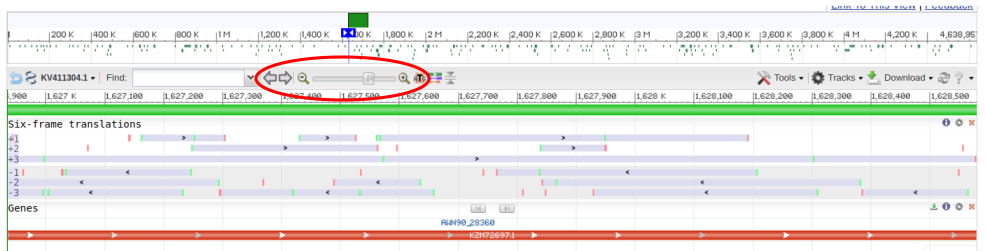

   7. We find the nocA sequence not far upstream of the nocC gene. A cross-check with the publication confirms that we have the right sequence.

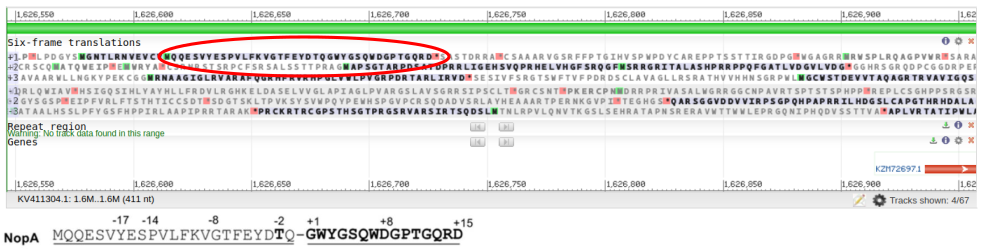

   8. To get the exact nucleotide start and stop positions, we right-click on the sequence to introduce two markers: one for the start (Marker 1) and one for the stop of the sequence (Marker 2). **Make sure to also include the stop codon.** Then, go to the Tools button and select “Markers”.

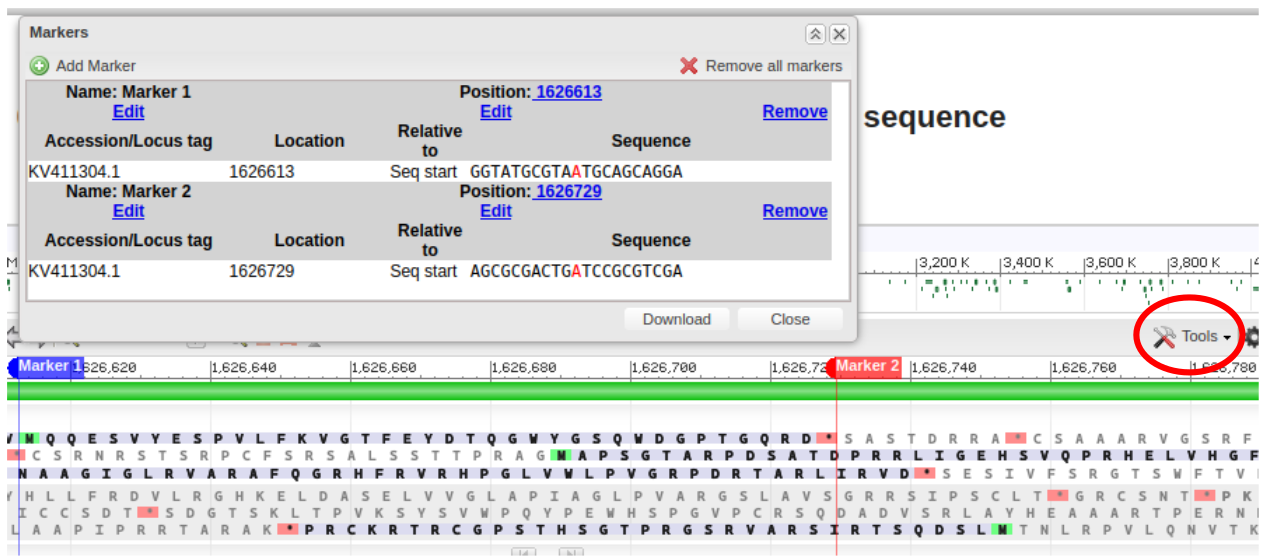

   9. Now, these coordinates (“Location”) can be used as input to declare the extra gene in the MIBiG submission portal. To verify that the correct location was chosen, the coordinates can be searched in the Genome Browser. For this, go to Tools -> Go To and enter the range (start and stop location, separated by a hyphen).

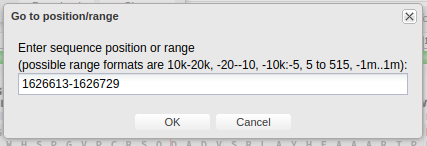

   10. Now, go back to GenBank.

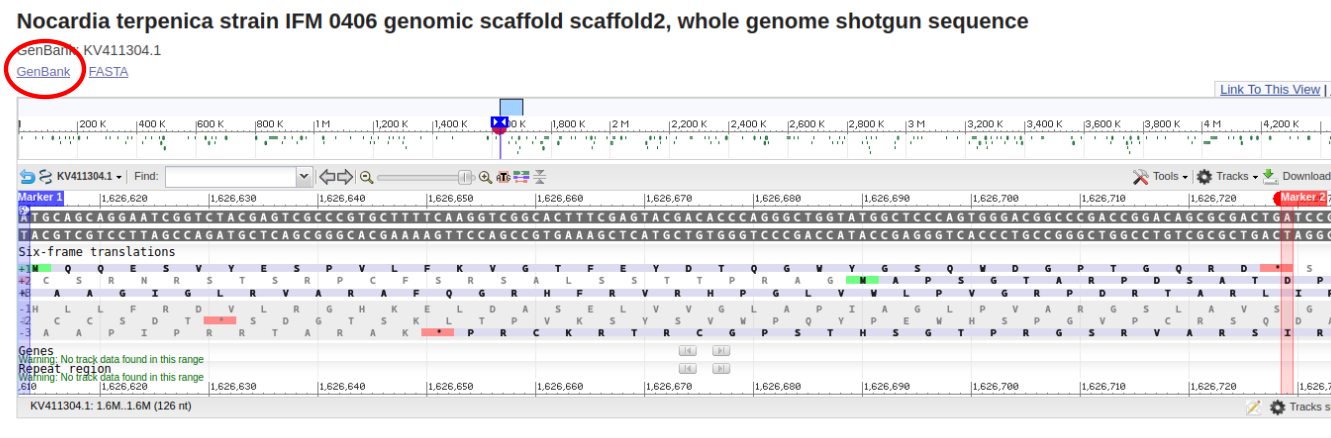

   11. Check if the URL has the right coordinates:

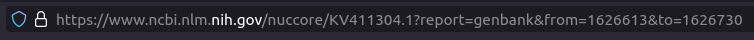

   12. Copy the resulting nucleotide sequence and translate it with a tool such as Expasy Translate (<https://web.expasy.org/translate/>). If everything went alright, the translation matches the amino acid sequence (including the stop codon).

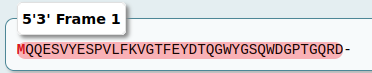


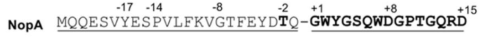

2. Add the subclass of the RiPP. Sometimes, this can be tricky, since new RiPP subclasses are frequently discovered, which may change the classification. Feel free to consult one of the RiPP Interest Group Coordinators or Reviewers with expertise in RiPPs. Current subclass options are: Unmodified, Atropopeptide, Biarylitide, Bottromycin, Borosin, Crocagin, Cyanobactin, Cyptide, Dikaritin, Epipeptide, Glycocin, Graspetide, Guanidinotide, Head-to-tail cyclized , Lanthipeptide, LAP, Lasso peptide, Linaridin, Methanobactin, Microcin, Microviridin, Mycofactocin, Pearlin, Proteusin, Ranthipeptide, Rotapeptide, Ryptide, Sactipeptide, Spliceotide, Streptide, Sulfatyrotide, Thioamidide, Thiopeptide, Other.
3. Provide the core sequence(s). Sometimes, this information must be transferred from a Figure in the original publication. Take care to not introduce any mistakes.
4. Provide the leader and/or follower sequence cleavage location. This information must be provided as genome coordinates (as explained above)
5. If applicable, add crosslink information and peptidase information (ID and cleavage recognition motif)

#### 3.2.4 Terpene BGCs

*Note: this description is still incomplete and may be expanded in the future. In case of questions, please contact the Terpene Interest Group Coordinators.*

For Terpene BGCs, add the following information:

1. Specify the subclass of the terpene: Diterpene, Hemiterpene, Monoterpene, Sesterterpene, Triterpene.
2. Specify the prenyltranserases
3. Specify the synthases/cyclases
4. Specify the precursor molecule
   1. DMAPP
   2. FPP
   3. GGPP
   4. GPP
   5. IPP

#### 3.2.5 Exotic/Other BGCs

*Note: this description is still incomplete and may be expanded in the future. In case of questions, please contact the Exotic BGCs Interest Group Coordinators.*

Exotic BGCs can be either Saccharides, or “Other” (summarizing non-canonical BGCs).

##### 3.2.5.1 Saccharide

1. Specify the subclass of the saccharide
2. Specify one or more glycosyltransferases
3. Specify one or more saccharide-producing subclusters

##### 3.2.5.1 Other

1. Specify the subclass of the BGC: aminocoumarin, cyclitol, other.
2. Specify additional details

### 3.3 How to add biological activity

Starting from MIBiG 4.0, only activities for purified molecules can be specified. Do not add activities for mixtures!

1. Specify the compound for which the activity should be added. Multiple compounds can be specified.
2. For each compound, add the activity. This is specified as an Assay. Under Property, you can select the biological activity from a drop-down list (Table 1.). Try to be as specific as possible, i.e. if a compound showed antibiotic activity against Gram-positive strains, please specify “anti-Gram-positive” instead of “antibacterial”.
3. Specify the relevant citations as DOI or PubMed IDs.

Table 1. Biological activity ontology used in MIBiG 4.0

| **Activity Type** | **Property** | **Activity Type** | **Property** |
| --- | --- | --- | --- |
|  |  |  |  |
| **Chemical properties** | Denitrificative | **Therapeutic properties** | Antibacterial |
|  | Emulsifier |  | Anti-Gram-negative |
|  | Flavor |  | Anti-Gram-positive |
|  | Fluorescent |  | Anticancer |
|  | Surfactant |  | Antineoplastic |
|  | Ionophore |  | Antitumor |
|  | Chalcophore |  | Antifungal |
|  | Lanthanophore |  | Antiinflammatory |
|  | Siderophore |  | Antioomycete |
|  | Zincophore |  | Antiparasidal |
|  | Odorous metabolite |  | Anthelmintic |
|  | Pigment |  | Antiplasmodial |
|  | Radical scavenging |  | Antimalarial |
|  |  |  | Antiprotozoal |
| **Cellular processes** | Adhesion |  | Anticoccidial |
|  | Biofilm |  | Antiproliferative |
|  | Cell differentiation |  | Antitubulin |
|  | Cell envelope |  | Antiviral |
|  | Cell wall |  | Herbicidal |
|  | Cell protectant |  | Antialgal |
|  | Cyst formation |  | Immunomodulatory |
|  | Exopolysaccharide |  | Immunosuppressive |
|  | Extracellular capsule |  | Insecticidal |
|  | Predation |  | Neuroprotective |
|  | Regulatory |  | Sodium channel blocking |
|  | Inducer |  | Toxic |
|  | Inhibitor |  | Cytotoxic |
|  | Proteasome inhibition |  | Cytostatic |
|  | Signalling |  | Dermatotoxic |
|  | Plant-defense signalling |  | DNA-interfering |
|  | Morphogen |  | Enterotoxic |
|  | Stress response |  | Hemolytic |
|  | Antioxidant |  | Hepatotoxic |
|  | Carbon storage |  | Irritant |
|  | Cold stress |  | Neurotoxic |
|  | Iron reducing |  | Phytotoxic |
|  | Nitrogen reduction |  | Tumor promoter |
|  | Osmolytic |  | Vesicant |
|  | UV protective |  |  |
|  | Swarming motility |  |  |
|  | Virulence factor |  |  |


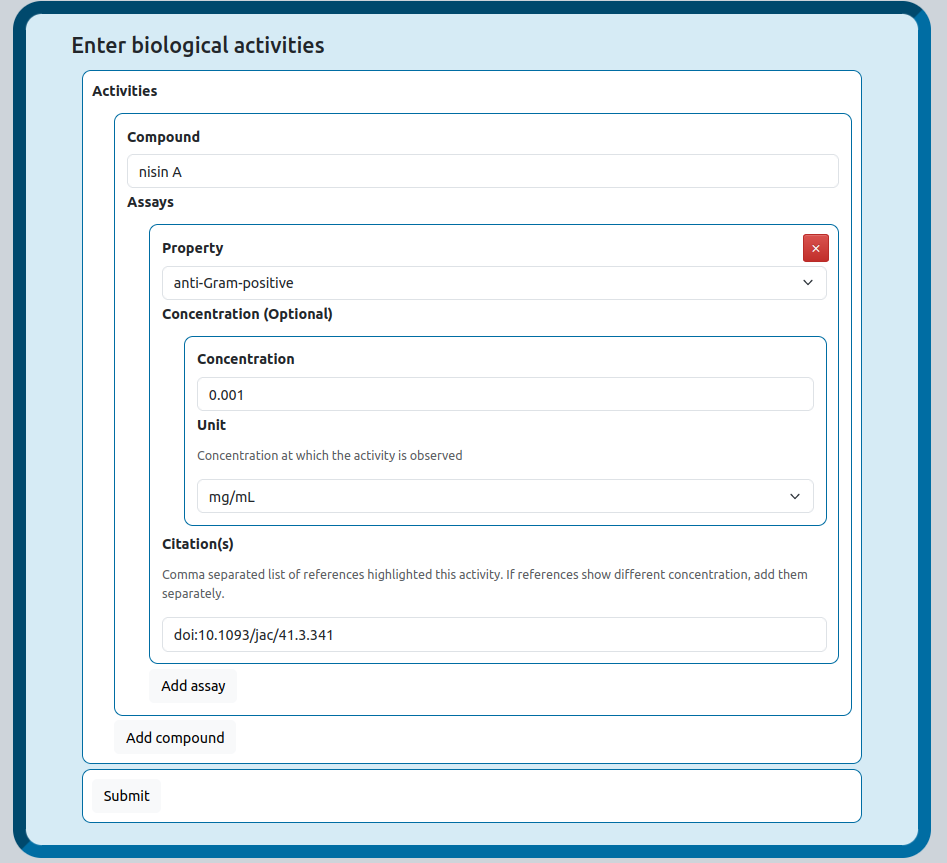


### 3.4 How to add the molecular structure(s)

Besides the molecular structure of the compound, other essential compound information can be added here as well.

1. Add the compound name and a chemical structure. This can be done in several ways. We suggest the following protocol (ranked from most to least preferred). Compare the structures visually to make sure they refer to the same molecule. Please pay particular attention to chiral centers and the constitution of lipid side chains.
   1. Add a Natural Product Atlas ID (no other details required) Note: NP Atlas only contains microbial compounds.
      1. Check NP Atlas for an entry, searching by compound name or structure. The easiest way to do this is using the Name field on the Basic Search page (<https://www.npatlas.org/search/basic>) which includes dynamic autofill for existing names.
      2. If you find a corresponding NPA entry, fill in the NPAID number (format NPA000001).
      3. If the compound reported in the publication shows no stereochemistry, but the NPAtlas-contained one has stereochemistry, this can be still accepted for MIBiG. NP biosynthesis is usually stereospecific, meaning that the resulting product is not a racemat. For example, if the authors show microcin J25 as planar structure (no stereocenters), but the NPAtlas has microcin J25 with stereocenters, the structure from NPAtlas can be taken. If in doubt, contact one of the Structures Coordinators.
   2. If no hits are found in NPA, add PubChem ID. Note: Reviewers pay special attention to make sure the PubChem structure matches the one in the paper.
   3. If no compound name is found or no hit is found using the compound name, add raw SMILES.
      1. Draw the structure in a chemistry drawing program such as MarvinSketch or ChemDraw.
      2. Copy the structure as a SMILES string. (Edit → Copy as → SMILES). For more detailed instructions, see [Advanced annotations for tailoring enzymes (MITE)](#kix.7vmv6bivuj8p).
      3. Also check NP-Atlas or PubChem for this molecule by searching based on the structure. The easiest way to do this is open the import panel in the structure plugin on the NPAtlas Basic Search page and paste in the SMILES string, then selecting the ‘Exact match’ radio button on the right of the structure pane. (Only record the ID and/or compound name if the hit matches exactly!). If a 100% hit is found, enter it in the sheet.
      4. If still no hit is found, enter the SMILES string in the form (preferably isomeric, recognizable by ‘@’ and/or ’\’ and/or ‘/’ in the SMILES string).
2. Provide the molecular (monoisotopic) mass and the molecular formula of the compound. This information is used to double-check the provided SMILES string for correctness.
3. Provide the experimental evidence (methods) that was/were used to characterize the compound(s). This information can usually be found by looking at the Methods and Materials section of the publication. Sometimes, a previous publication has characterized the compound, and needs to be referenced too. Often, multiple evidence types were used, and therefore, multiple options can be added. Accepted evidence types are
   1. Total synthesis
   2. X-ray crystallography
   3. Chemical derivatization (e.g. Marfey’s analysis)
   4. NMR
   5. MS/MS (tandem mass spectrometry)
   6. Mass spectrometry
4. (Optional) Provide the chemical class of the compound. MIBiG 4.0 uses a set biosynthesis-informed high level compound classification, based on the standard textbook **Medicinal Natural Products by Paul M Dewick** [(5)](https://paperpile.com/c/TLintO/7iLo) (Table 2). Multiple chemical classes can be assigned. If in doubt, consult with one of the Coordinators of the Chemical Structures Interest Group.
5. (Optional) Provide cross-references to databases in which the compound can be found. Please provide respective compound IDs. Cross-links can be made to (in alphabetical order):
   1. PubChem (<https://pubchem.ncbi.nlm.nih.gov/>)
   2. ChEBI (<https://www.ebi.ac.uk/chebi/>)
   3. CHEMBL (<https://www.ebi.ac.uk/chembl/>)
   4. ChemSpider (<http://www.chemspider.com/>)
   5. CyanoMetDB (<https://zenodo.org/records/4562688>)
   6. GNPS (<https://gnps.ucsd.edu/>)
   7. Lotus (<https://lotus.naturalproducts.net/>)
   8. NPAtlas (<https://www.npatlas.org/>)

Table 2. The chemical classification system used by MIBiG 4.0

| **Chemical Class** | **Chemical Subclass** | **Chemical Class** | **Chemical Subclass** |
| --- | --- | --- | --- |
|  |  |  |  |
| **Alkaloid** | Amination reaction-derived | **Shikimic acid-derived** | Aromatic amino acid/simple benzoic acid |
|  | Anthranilic acid-derived |  | Aromatic polyketide |
|  | Arginine-derived |  | Phenylpropanoid |
|  | Guanidine-derived |  | Terpenoid quinone |
|  | Histidine-derived |  |  |
|  | Lysine-derived | **Acetate-derived** | Alkylresorcinol/phloroglucinol polyketide |
|  | Nicotinic acid-derived |  | Chromane polyketide |
|  | Ornithine-derived |  | Cyclic polyketide |
|  | Peptide alkaloid |  | Fatty acid |
|  | Proline-derived |  | Fatty acid derivate |
|  | Purine alkaloid |  | Linear polyketide |
|  | Serine-derived |  | Macrocyclic polyketide |
|  | Steroidal alkaloid |  | Naphthalene polyketide |
|  | Tetramate alkaloid |  | Polycyclic polyketide |
|  | Terpenoid-alkaloid |  | Polyether polyketide |
|  | Tryptophan-derived |  | Xanthone polyketide |
|  | Tyrosine-derived |  |  |
|  |  | **Peptide** | Beta-lactam |
| **Isoprene-derived** | Atypical terpenoid |  | Depsipeptide |
|  | Diterpenoid |  | Diketopiperazine |
|  | Hemiterpenoid |  | Glycopeptide |
|  | Higher terpenoid |  | Glycopeptidolipid |
|  | Iridoid |  | Linear |
|  | Meroterpenoid |  | Lipopeptide |
|  | Monoterpenoid |  | Macrocyclic |
|  | Sesquiterpenoid |  |  |
|  | Steroid | **Glycolysis-derived** | Butenolides |
|  |  |  | γ-Butyrolactones |
| **Carbohydrates** | Monosaccharide |  | Tetronic acids |
|  | Oligosaccharide |  |  |
|  | Polysaccharide | **Other** | Lactone |
|  | Nucleoside |  | Ectoine |
|  | Aminoglycoside |  | Furan |
|  | Liposaccharide |  | Phosphonate |
|  | Glucosinolate |  |  |
|  | Glucosinolate |  |  |

### 3.5 How to add gene annotations:

Gene annotations are an important part of MIBiG. While not mandatory, they provide information on functions of genes and aid in the interpretation of genomic data. Without gene annotations, interpreting BGCs can be very challenging.

#### 3.5.1 Basic gene annotations

For each gene in a BGC, annotation about its function can be added. This information can be selected from a drop-down menu, but also provided in a free text field. Valid drop-down options are: Activation / processing, Maturation, Precursor, Precursor biosynthesis, Regulation, Resistance/immunity, Scaffold biosynthesis, Tailoring, Transport, Other. Adding basic gene annotation is already greatly helping with BGC interpretability. Make sure that the function of the gene has been experimentally verified and not only predicted based on homology.

#### 3.5.2 Advanced annotations for tailoring enzymes/maturases (MITE)

Alternatively, more specific and detailed information about tailoring enzymes/maturases can be added using the Minimum Information about a Tailoring Enzyme (MITE) data standard [REF TBA]. This recently developed data standard allows to store information about the reaction(s) performed by a tailoring enzyme/maturase, including the substrate specificity, and example substrate-product pairs. MITE focuses on post-scaffold synthesis tailoring enzymes/maturases specifically. These enzymes do not need to be necessarily clustered with the BGC.

**Nota bene:** In MITE, the substrate specificity and enzymatic reaction are stored as a so-called **reaction SMARTS**, a line representation of the transformation of the substrate-product pair. The most convenient way of creating such a reaction SMARTS is by drawing it in a chemistry drawing program. We recommend MarvinSketch by ChemAxon, which is free for individual, academic and non-commercial use and available for Windows, Mac, and Linux (<https://download.chemaxon.com/marvin>). The protocol was written assuming the use of MarvinSketch. Furthermore, MarvinSketch allows export of reaction **CXSMARTS**, which have expanded structure representation functions, explained in more detail in the respective section of the protocol. Of course, use of MarvinSketch is not mandatory and reaction SMARTS can be created in various ways (even written manually!). However, reaction SMARTS not created by MarvinSketch must be at least RDKit-compatible to be accepted by MITE.

Perform the following steps to add the MITE information:

1. From the publication, determine the substrate specificity, regioselectivity and the reaction that the enzyme performs. Make sure that the enzyme matches the one that is in the MIBiG entry. Either follow the tutorial steps below or watch the following video: <https://youtu.be/WJDR_vQMY-s>
   1. In your chemistry drawing program, start drawing the substrate (sub)structure that is going to be modified (in this protocol, all steps are shown using MarvinSketch). Also make sure to correctly depict the stereochemistry. Some enzymes are very specific with regard to their substrate, and large and specific substrate/product structures need to be drawn. Other enzymes are very promiscuous and can therefore also work on a more generic substrate. This data can often be found in the paper, and it is essential to capture this information accurately. Below, a hypothetical peptide was drawn (GAXFE, where X indicates a non-specified amino acid, represented by a glycine due to its lack of residue).

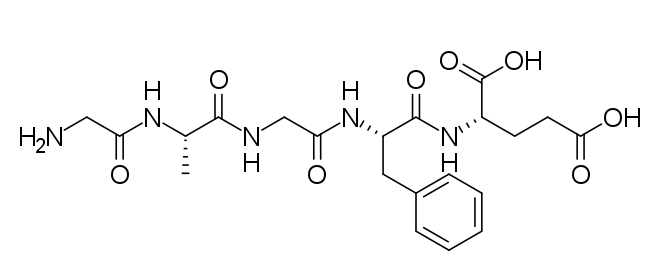

   2. Next, the chemical structure must be turned from the Kekulé form into the aromatic form, else, the aromaticity information is not properly encoded. In MarvinSketch, select the structure, and in the menu, click **Structure** -> **Aromatic Form** -> **Convert to Aromatic Form**.

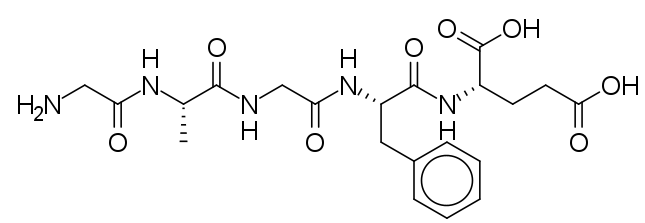

   3. Next, map the atoms (assign index numbers to them). In MarvinSketch, select the structure, and in the menu, select **Structure** -> **Mapping** -> **Map Atoms**. This will assign an unique index number to all atoms.

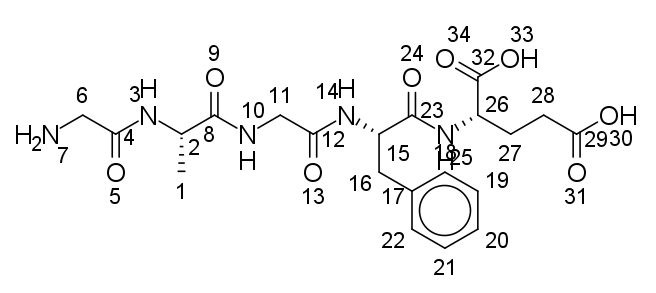

   4. Next, copy the structure and draw the reaction arrow. In MarvinSketch, select the structure, and in the menu, select **Edit** -> **Copy** (or use the Ctrl+C key combination). Then, select the reaction arrow from the left-hand side toolbar, and draw an arrow from left to right.

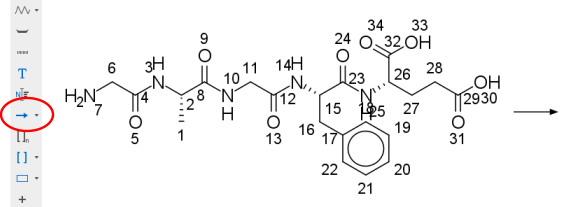

   5. Next, paste your previously copied substrate on the product side (the right-hand side of the arrow).

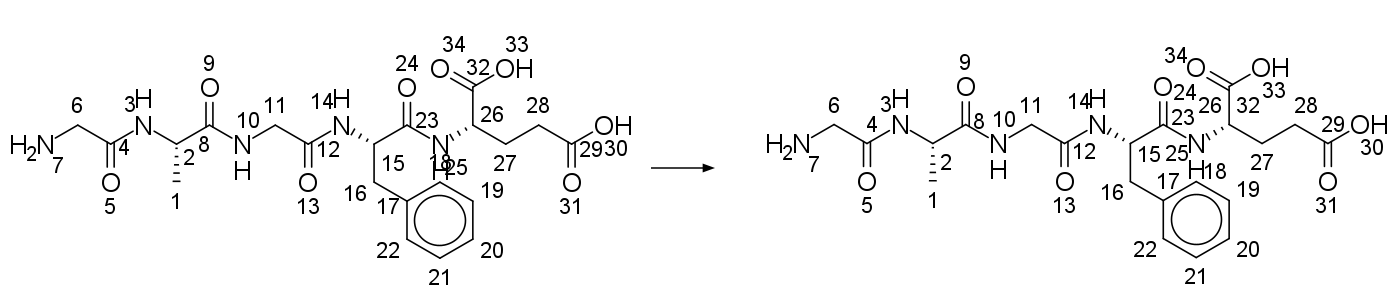

   6. Next, draw the changes that are introduced by the enzymatic reaction. If this introduces any new atoms, they also have to be mapped. This can be done by selecting the newly added atom, right-clicking on the canvas, and selecting **Map** -> **M…** -> adding a so-far **unused** number. If this concludes the drawn reaction, go on to **step i).** In our hypothetical example, we assume that the enzyme introduces both a **macrolactam cyclization** and a **chlorination**. The macrolactam cyclisation leads to a loss of water, which does not have to be accounted for. However, we have to map the new chlorine atom, and we give it the unused index *‘40’*.

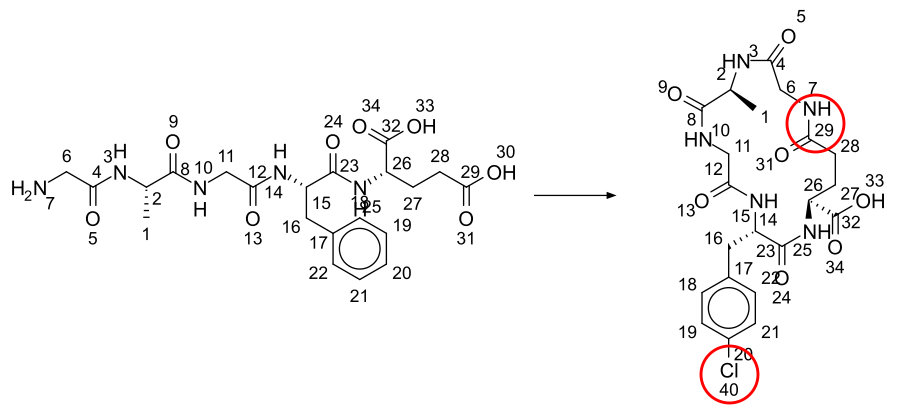

   7. (**Optional**: *Position Variation Bonds*) With **MarvinSketch**, we can use specific functionality to assign additional information to the reaction. One functionality is adding *Position Variation Bonds*. These specify variable locations for a functional group (e.g. variable chlorination on an aromatic ring). To add Position Variation Bonds select the atoms where the optional bond will be located. Then, in the menu, go to **Structure** -> **Add** -> **Position** **Variation Bond.** This will create a free floating bond and a gray border around the previously selected atoms, indicating the atoms to which the functional group will be applied. Now, add the desired atom or functional group to the outward side of the floating bond. As before, add atom mappings to the added atom/functional group. In our example, we want to indicate that there are multiple chlorinations on the phenol-ring: one in the *para*-position, and either one in the *ortho*- or *meta*-position.

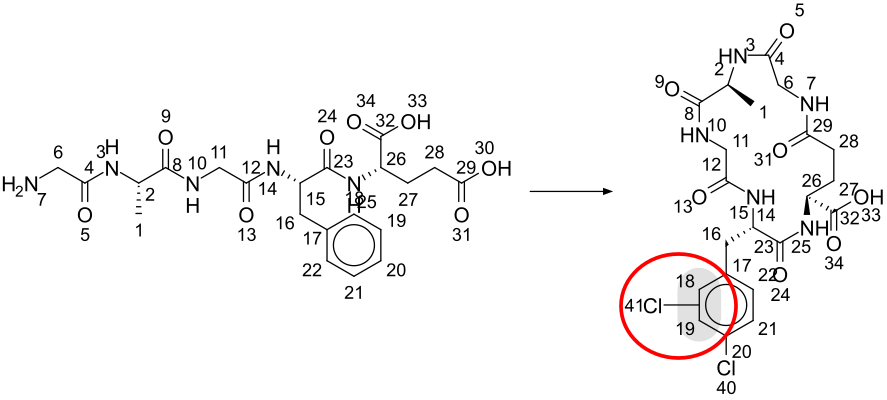

   8. (**Optional**: *Frequency Variation*) With **MarvinSketch**, we can use specific functionality to assign additional information to the reaction. One functionality is adding *Frequency Variation*. This allows specifying certain repeating elements (e.g. an aliphatic carbon chain of variable length). To add Frequency Variation, select the atom(s) where the Frequency Variation label should be applied. Then, go to **Structure** -> **Group** -> **Frequency Variation**. In the pop-up menu, set *“type”* to *"Repeating unit with repetition ranges"*. Set *"repetition range"* to a fixed number of repetitions (e.g. **2** to repeat units twice) **or** a range (e.g. **2-3** to repeat units twice or thrice). Set *"Polymer repeat pattern"* to *"head-to-tail"* (no other pattern is supported) and *"bracket style"* to "*square[]*". In our example, we want to indicate that the macrocyclization can happen with the N-terminal glycine and either a C-terminal aspartic or glutamic acid (one or two C-atoms, respectively).

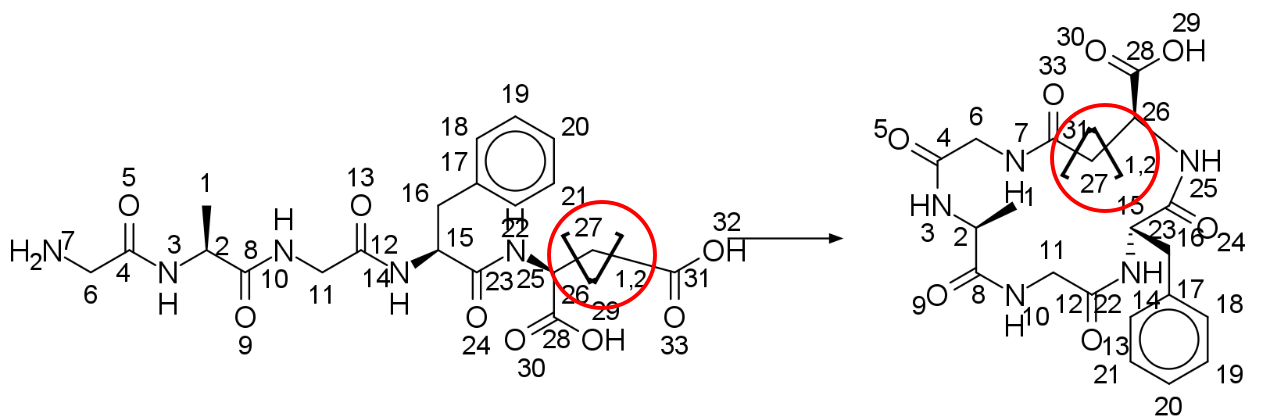

   9. Finally, to export the reaction, select substrate, product, and the reaction arrow, right-click on canvas, and select **Copy As** -> **ChemAxon SMARTS (CXSMARTS),** which stores the SMARTS string in the clipboard. Now, you can paste the string into the corresponding field in the MIBiG Submission Portal. If you want to verify if the SMARTS was exported correctly, you can also try to paste it on the canvas. If everything went right, you should see the complete reaction.
2. After the reaction SMARTS/CXSMARTS was added, some additional information needs to be specified: the literature reference, evidence, and any database crosslinks; is the reaction iterative (i.e occurs multiple times exhaustively); does it contain Frequency Variation of Position Variation Bonds (see above); and finally, are there any explicit hydrogen atoms to specify. By default, SMARTS strings **do not** preserve hydrogen atoms. For example, to indicate a primary amine, two hydrogens need to be explicitly specified. In our previous example, the primary amine of the N-terminal glycine needs to be specified; else, the SMARTS string would also match a pattern inside a longer peptide chain. Further, also the hydrogens on the alpha-carbon of Gly1 need to be specified explicitly; else, the pattern would match any amino acid. For the X (any) amino acid in position three, we do not specify any explicit hydrogens - this way, this position will match any amino acid.

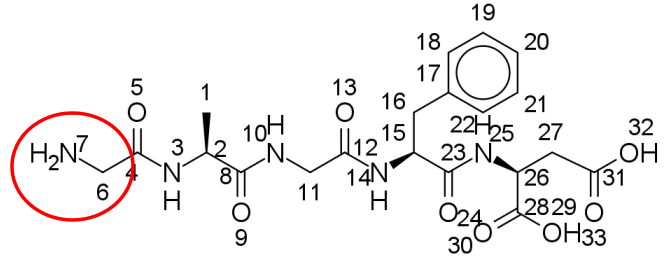

3. Next, to validate the reaction SMARTS, one or more **substrate - product pairs** need to be specified. These structures must be specified as SMILES strings. The substrate - product pair can be either a balanced, authentic reaction, or also just an example reaction (e.g. when the exact substrate and/or product is not known).
   1. Draw the substrate (or the substructure, if the exact substrate is not known) using any chemistry drawing tool. If the substrate - product pair should be balanced, also add any supplementary reaction partners or co-factors. Select all molecules, right-click on canvas, and select **Copy As** -> **ChemAxon SMILES (CXSMILES),** which stores the SMILES string in the clipboard. Now, you can paste the string into the corresponding field in the MIBiG Submission Portal. If you want to verify if the SMILES was exported correctly, you can also try to paste it on the canvas. If everything went right, you should see all substrates. In our example, we drew the peptide GAWFD, substituting the “any” amino acid in the third position with a tryptophan.

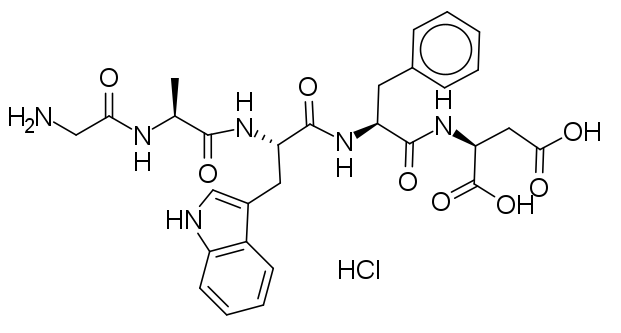

   2. Draw the product (or the substructure, if the exact product is not known) using any chemistry drawing tool. If the substrate - product pair should be balanced, also add any supplementary reaction partners or co-factors. Export the SMILES string as described in point 3a. Multiple products can be specified, if necessary

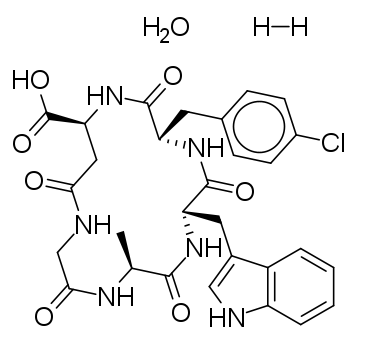

   3. Next, some additional information needs to be specified: is the reaction balanced (i.e. is it stoichiometrically balanced); is the reaction authentic (i.e. not only substructures); is the reaction describing an intermediate (i.e. not the reaction step that leads to a mature product); any database cross-references and finally, a literature reference and the evidence for the reaction pair.
   4. If necessary, multiple reaction pairs can be described.
4. Next, the term that best describes the tailoring/maturation reaction must be selected from the list of tailoring reactions. Valid terms are: Acetylation, Acylation, Amination, Biaryl bond formation, Carboxylation, Cyclization, Deamination, Decarboxylation, Dehydration, Dehydrogenation, Demethylation, Dioxygenation, Epimerization, FADH2 supply for chlorination, Glycosylation, Halogenation, Heterocyclization, Hydrolysis, Hydroxylation, Macrolactam formation, Methylation, Monooxygenation, Oxidation, Phosphorylation, Prenylation, Reduction, Sulfation, Other. Multiple terms can be specified.
5. Additionally, some information about the tailoring enzyme/maturase must be specified. This includes the commonly used name of the protein and an optional description; cross-references to UniProt and/or NCBI GenPept as well as the primary literature reference. Also, any auxiliary enzymes that are co-forming the maturation machinery can be specified with name and database cross-references (e.g. in case of microcin J25, both McjB and McjC are required for the lasso peptide macrolactam formation, so for an entry of McjB, McjC needs to be specified as auxiliary enzyme, and vice versa).
6. Finally, a click on the “Validate” button checks if the reaction SMARTS/CXSMARTS is correctly formatted and if it can create the specified product(s) from the specified substrate. A message will indicate the outcome of the validation.

#### 3.5.3 FAQ MITE

- Q: What is the scope of MITE and which enzymes can be added? A: MITE accepts data on post-scaffold biosynthesis tailoring enzymes. For NRPS, PKS, this would be any structure-modifying enzyme after the mega-synthase. For terpenes, this would be any enzyme acting post-terpene cyclase. For ribosomal peptides, this would be any modification happening post-ribosomally.

## Section 4: Instructions for Reviewers

The MIBiG 4.0 Annotathons see the introduction of the role of Reviewer. Reviewers are topic matter experts who scrutinize newly created or modified entries for plausibility and scientific soundness. Reviewers may request revisions to entries that contain erroneous or incorrect data, and provide constructive feedback for their improvement.

Annotathon reviewers are acknowledged in MIBiG entries by having their names displayed on the MIBiG website, and a separate acknowledgment in the MIBiG publication. Opting out of the name display in MIBiG/website is possible during registration. However Trello Cards will still display the Reviewer's name/ID.

Reviewers are expected to:

- Ensure data quality and scientific correctness.
- Scrutinize newly created/modified entries.
- Fix small mistakes themselves (rule of thumb: should take less than 5 minutes).
  - E.g. typos, wrong chirality, wrong biosynthetic (sub)classes, etc.
- Flag problematic entries for revision (not fixing the whole entry).
  - Communicate with Contributor/Coordinators if necessary during the revision process.

### 4.1 Prerequisites

Reviewers who work on chemical structures or on the Minimum Information about a Tailoring Enzyme (MITE) entries will need to install a chemistry drawing program (e.g. Chemaxon MarvinSketch) for visualization of SMILES and reaction SMARTS strings.

### 4.2 The Review Process

Once a (group of) Contributor(s) has/have finalized generating or modifying an entry, the relevant Trello Card is placed from the **In Progress** in the **To Review** List. A Reviewer will then assign themselves to this card, and move it to the **In Review** List, to indicate that the entry is being reviewed/worked on. Biosynthetic Class and Annotation Labels will help Reviewers select which cards/entries best fit their expertise.

Using the **MIBiG UID** available in the card description, a Reviewer will be able to retrieve the entry in the MIBiG Submission Portal and start reviewing. During this review process, a Reviewer will essentially re-trace the steps of the contributor(s), making use of the submitted citations to ensure data quality and scientific correctness of each (and all) of the fill-out fields. Reviewers will also perform quick searches to confirm that the label “Not currently available” (e.g. for a missing compound structure) has been correctly filled out. If a Reviewer finds any small mistakes that take no more than a few moments to fix, and which may require only a quick and easy exchange with a contributor, they are expected to fix such mistakes themselves.

A Reviewer may choose to review an entire entry, or a section of an entry, in which case the Reviewer will tick-off the relevant Trello card checklist item, add a brief comment in the activity log, and unassign themselves from the card again. Another Reviewer can then assign themselves to the card and proceed with reviewing another section of the entry. This process repeats until all checkboxes are ticked, at which point the card can be moved **by the Reviewer** to the List **Done**.

### 4.3 Frequently observed Problems

- Are the locus coordinates correct? Can the locus be found in GenBank?
- Is the complete BGC represented by the GenBank entry (all genes present)?
- Does the referenced locus contain any genes which are not part of the BGC? Are these genes appropriately labeled (excluded)? For example, the “[albomycin biosynthetic gene cluster](https://www.ncbi.nlm.nih.gov/nuccore/JN252488.1)” record contains seven additional ORFs that are not part of the cluster in the [publication](https://www.jbc.org/article/S0021-9258(23)00215-6/fulltext). Such spurious genes should be “removed” from the annotation.

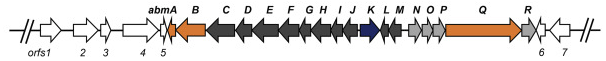

- Is the strain identifier correct?
- Are chemical structures correct?
- Are gene annotations correct?
- TBA

### 4.4 The Revision Process

If at any point during the review major errors/issues are found, Reviewers will move the card into the **Revision** List, and start a revision process. Here, a Reviewer will request revision with specific feedback from the relevant Contributor(s) and if necessary a Coordinator(s) by tagging the person(s) on the Trello card, and reaching out via Slack/Zoom/Email. The Reviewer will also add a comment in the activity log describing the reasoning behind the need for a revision. The responsible person can be identified by inspecting the activity log.

A dedicated Slack channel is available to discuss difficult cases and leverage the large contributor/reviewer/coordinator network’s support for solving annotation issues. Please always be polite and objective, but definitive; rebuttals are not planned. If necessary, escalate to the MIBiG Organizational Team.

Once the issue has been addressed/fixed, if there are sections of the entry that still need to be reviewed, the currently assigned Reviewer will either complete the review, or reach out to a Coordinator that can help assign a different Reviewer so that the review process is finalized. Once this is done, the card is moved to the **Done** List.

## Section 5: Frequently Asked Questions

#### What to do if a paper lacks even the essential information to create an entry?

Sometimes while creating new entries, it becomes clear that the publication lacks essential data to create an entry. This can be due to a missing genome accession, an embargoed genome, or other essential information. In such cases, in the Trello board, the card should stay in the “IN PROGRESS” column, the label **“Critical Error”** should be added, and one of the Interest Group Coordinators notified. They will decide how to proceed further - closing the entry, requesting additional information from the authors, or resolving the issue in another way.


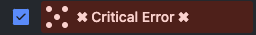


Figure X: card label indicating a critical issue with a card or entry.

#### Does any specific software need to be installed before the annothatons?

For basic functionality, a contemporary web browser such as Mozilla Firefox or Google Chrome is sufficient. For drawing chemical structures and adding advanced tailoring enzyme information (MITE), a chemistry drawing program is required. We recommend MarvinSketch by ChemAxon, which is free for individual, academic and non-commercial use and available for Windows, Mac, and Linux (<https://download.chemaxon.com/marvin>). Most of this protocol was written assuming the use of MarvinSketch.

#### Multiple BGCs from a single paper

Sometimes, a publication characterizes more than one BGC from an organism, or multiple BGCs from different organisms. For each BGC described by such a publication, a separate MIBiG entry should be prepared - that means, also creating separate cards in the Trello board.

#### No compound name

Sometimes, publications do not name compounds with trivial names (e.g. erythromycin A) but only with numbers and/or chemical nomenclature (IUPAC) names. In such cases, the IUPAC name should be added. If no such name is available, the name of the compound can be stated as “Not named”. However, please also get in touch with one of the coordinators for chemical structures which can help with generating a IUPAC chemical name.

#### Minimal Evidence for MIBiG?

The consensus is that **at least** a corresponding signal using mass spectrometry (or an equivalent analytical technique) should have been observed (**“Correlation between genomic and metabolomics information”**). Many papers only find BGCs similar to published ones using computational methods and report those without ever observing any evidence of the compounds. **Such publications do not satisfy the MIBiG experimental data requirements.**

#### What if a paper lacks e.g. A-domain specificities?

This is perfectly fine – there is some minimum information required to create a new MIBiG entry, and everything else is optional. Ideally, each MIBiG entry would have a complete set of information, but we know that this is not possible since not all BGCs have been fully experimentally characterized.

#### Automated way of submitting large in-house datasets?

If you want to submit a large dataset, it is best to get in touch with us to find a solution.

#### How long does it take to create one MIBiG entry?

This strongly depends on the underlying publication and the amount of information extracted, but on average, it can be estimated to be half an hour to one hour per BGC/Publication.

#### Work outside of annotathon times?

Work outside the annotathon times is fine, but people should participate at least during the first annotathon to be able to ask questions about the submission system. Ideally, they would be online during part of the other annotathons too, especially Reviewers who may need to connect with Contributor(s) and/or Coordinators with regard to revisions.

#### Can Reviewers also create entries?

Yes, especially at the beginning, there may not be many entries to review, so it is perfectly fine to also create entries while having the role of Reviewer. However, once the first entries have been created, the priority should be on reviewing. Entries created by a reviewer must still be placed in the “to review” column; do not “review” your own entries.

#### What is the deadline for creating/reviewing entries?

The general deadline on activities on MIBiG 4.0 is **June 1st 2024.**

#### How to treat papers that are currently under submission/in revision?

Information from papers currently under submission or in revision is possible. It is also possible to submit data from preprints. Short embargo periods are also allowed. However, keep in mind that entries (even embargoed ones) will be seen by at least one reviewer and are therefore not completely confidential.

#### On the Trello board, is each card a BGC OR a publication?

On the Trello board, **each card is considered one BGC**. One publication may describe multiple BGCs at the same time. Therefore, it is fine that multiple cards have the same publication as reference.

#### How to deal with partially clustered/non-clustered metabolite pathways?

Individual satellite genes or subclusters are generally accepted and can be referenced with multiple loci, belonging to the same MIBiG entry. **Completely unclustered pathways are outside the scope of MIBiG.** MIBiG is primarily a repository of biosynthetic gene clusters, which are defined to be clustered in physical vicinity in the genome.

#### What if a BGC misses some genes because it lies on a contig edge?

If the missing genes can be located somewhere else in the genome, they can be added as satellite genes (see the question *“How to deal with partially clustered/non-clustered metabolite pathways?”*). If the missing genes cannot be located, the MIBiG entry is considered “incomplete”, but can still be created.

#### What if a BGC product is glycosylated, but the BGC lacks a glycosyltransferase?

If the glycosyltransferase can be located, it can be added as a satellite gene (see the question *“How to deal with partially clustered/non-clustered metabolite pathways?”*). If the glycosyltransferase cannot be located, the MIBiG entry is considered “incomplete”, but can still be created.

#### For NRPS A-domain specificity, which amino acid should be added?

There are two fields: one for the substrate that the A-domain loads, and another for the module that introduces a monomer in the structure.

#### Why not use large language models to extract data from publications?

We have experimented with this approach but currently, it is very error-prone. It works well to select articles to read, but fails to extract the actual annotations.

#### For MITE, are two homologous genes/enzymes two different entries?

Yes, in MITE, there is one entry for each enzyme. It follows the MIBiG logic of having different entries for homologous BGCs. Therefore: one enzyme -> one MITE entry.

#### Is it worth adding an already present BGC from a different organism?

While not a priority, it can still be interesting. For example, there are four bottromycin BGCs in MIBiG, all from different organisms.

# Bibliography

[1. Medema,M.H., Kottmann,R., Yilmaz,P., Cummings,M., Biggins,J.B., Blin,K., de Bruijn,I., Chooi,Y.H., Claesen,J., Coates,R.C., *et al.* (2015) Minimum Information about a Biosynthetic Gene cluster. *Nat. Chem. Biol.*, **11**, 625–631.](http://paperpile.com/b/TLintO/78JV)

[2. Terlouw,B.R., Blin,K., Navarro-Muñoz,J.C., Avalon,N.E., Chevrette,M.G., Egbert,S., Lee,S., Meijer,D., Recchia,M.J.J., Reitz,Z.L., *et al.* (2023) MIBiG 3.0: a community-driven effort to annotate experimentally validated biosynthetic gene clusters. *Nucleic Acids Res.*, **51**, D603–D610.](http://paperpile.com/b/TLintO/T5pp)

[3. Dell,M., Dunbar,K.L. and Hertweck,C. (2022) Ribosome-independent peptide biosynthesis: the challenge of a unifying nomenclature. *Nat. Prod. Rep.*, **39**, 453–459.](http://paperpile.com/b/TLintO/ekQ3)

[4. Saad,H., Majer,T., Bhattarai,K., Lampe,S., Nguyen,D.T., Kramer,M., Straetener,J., Brötz-Oesterhelt,H., Mitchell,D.A. and Gross,H. (2023) Bioinformatics-guided discovery of biaryl-linked lasso peptides. *Chem. Sci.*, **14**, 13176–13183.](http://paperpile.com/b/TLintO/yEIe)

[5. Dewick,P.M. (2009) Medicinal Natural Products: A Biosynthetic Approach Wiley.](http://paperpile.com/b/TLintO/7iLo)

1. Including archaeal, for simplification [↑](#footnote-ref-0)
